# Biomimetic Material Selection for Therapeutic Microneedles: An Analytic Hierarchy Process-Based Multi-Criteria Evaluation

**DOI:** 10.3390/polym18121456

**Published:** 2026-06-11

**Authors:** Hussain F. Abualkhair, Fahad Sulaiman Obaid, Mohammed Alquraish, Faisal Khaled Aldawood

**Affiliations:** 1 Department of Industrial Engineering, College of Engineering, Taif University, P.O. Box 11099, Taif 21944, Saudi Arabia; 2 Department of Mechanical Engineering, College of Engineering, University of Bisha, P.O. Box 551, Bisha 61922, Saudi Arabia; 3 Department of Industrial Engineering, College of Engineering, University of Bisha, P.O. Box 551, Bisha 61922, Saudi Arabia

**Keywords:** analytic hierarchy process, biomedical application, drug-delivery systems, medical devices, microneedle, multi-criteria decision making, polymeric materials, therapeutic drug delivery

## Abstract

Microneedles are a new technology in transdermal drug delivery that allows for the pain-free administration of drugs. Recently, these microneedles have gained popularity compared to traditional injections. Nevertheless, the selection of the most suitable materials is a significant issue, requiring a systematic analysis of various performance parameters. This paper developed a multi-criteria decision-making model based on the analytic hierarchy process (AHP) to systematically evaluate four primary material types for therapeutic microneedle applications: polymers, metals, ceramics, and silicon. The researchers defined five performance criteria, ranked by importance: biocompatibility (48.8%), mechanical properties (25.3%), manufacturability (15.8%), cost-effectiveness (6.6%), and compatibility with different types of microneedles (3.5%). The validity of the framework was established using the TOPSIS and ELECTRE methods, which showed strong agreement in the rankings, and a sensitivity analysis revealed that the rankings did not change with a ±20% variation in the parameters in 95% of the cases. The outcomes indicated that polymers are the most suitable, with the highest global priority score (38.3%), and they are good in biocompatibility (53.0% local priority), manufacturability (53.3%), and relative cost advantages (62.2%), though medical-grade polymer costs remain substantial. Metals were placed second (31.8%) due to their better mechanical properties (50.3%), followed by ceramics (17.6%) and silicon-based materials (12.3%). The framework offers clear decision guidelines: polymers for dissolving microneedle systems and controlled drug release applications; metals for precise liquid delivery devices; ceramics for specialized pharmaceutical uses that require extreme chemical compatibility; and silicon for research applications requiring precise geometries.

## 1. Introduction

Microneedles (MNs) represent a significant step in changing the methods of drug delivery via the transdermal route, since they offer a remarkable approach that circumvents the limitations of conventional injectable drugs and topical administration [1,2]. These microscopic needles (usually 25 to 1500 μm long and 50 to 250 μm wide) are designed to penetrate the outermost layer of skin, the stratum corneum, but not the pain-sensing dermis, making drug administration painless [3,4]. There are many cases where this technology could be used therapeutically, including administering vaccines [5], delivering drugs such as insulin to treat diabetes [6], producing hormones for hormone replacement therapy [7], treating cosmetic conditions [8], and collecting small biosamples non-invasively [9]. The market size of microneedles worldwide has been growing exponentially and is estimated to exceed 2.5 billion dollars in the current year (2025), owing to the rising demand for painless drug-delivery tools and the increasing prevalence of chronic diseases that require frequent drug administration [10]. According to the current trend, microneedle technology offers commercial opportunities and clinical advantages, especially in enhancing the compliance in the injection-based medical field [11,12].

The principle of microneedles is that they can pierce the main skin membrane without eliciting nerve responses. The stratum corneum is a layer of dead cells, known as keratinocytes and intercellular lipid matrix, that is 10 to 20 μm thick, providing a rigid coating that does not allow the drug to enter the skin [13]. Microneedles must be mechanically strong enough to penetrate this layer, but at the same time strong enough not to break during insertion, drug delivery, etc. [14]. Their painless administration is explained by their shallow penetration depth, which avoids the dermis, the area containing nociceptors and nerve endings that mediate pain [15]. Current microneedle technologies are divided into several categories based on therapeutic applications, each with distinct material and performance requirements. Solid microneedles must be pre-treated to form microchannels, after which topical drugs are loaded [16]. Coated microneedles have a drug coating on their surfaces for immediate release when they come into contact with the skin [17]. Microneedles, composed of biocompatible and biodegradable materials, dissolve in the skin after drug delivery, thereby eliminating the problems associated with sharp waste [18]. Hollow microneedles with internal channels have been developed as a controlled liquid drug-delivery system, similar to microneedles used as hypodermic needles for insulin delivery and other liquid therapeutics [19]. Solid piercing with hydrogel expansion for controlled drug release involves swellable microneedles that combine a solid-piercing delivery system with drug delivery via hydrogel expansion [20].

### 1.1. Working Principle of Microneedles and Advantages over Conventional Drug Delivery

**Figure 1:**
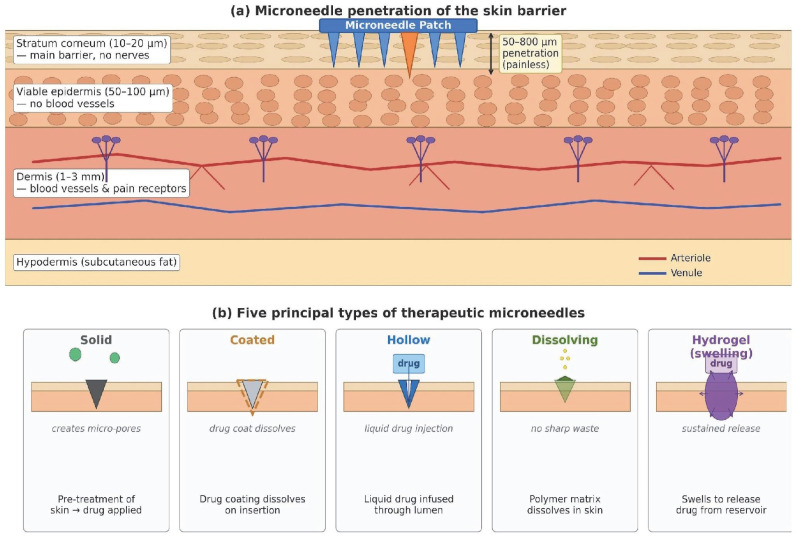
Working principle of microneedles for transdermal drug delivery. (**a**) Cross-sectional schematic showing penetration of the stratum corneum and viable epidermis while remaining shallow of the dermal nociceptors and blood vessels, accounting for painless administration. (**b**) The five principal therapeutic microneedle architectures—solid, coated, hollow, dissolving, and hydrogel-forming (swelling)—each tailored to different drug-delivery modes.

Microneedle arrays exploit the layered architecture of human skin to deliver therapeutic agents in a minimally invasive, painless manner. The outermost stratum corneum (10–20 μm) constitutes the principal diffusion barrier but is itself avascular and devoid of sensory innervation; the underlying viable epidermis (50–100 μm) is similarly free of blood vessels, while nociceptors and capillaries reside deeper, within the dermis (1–3 mm) [1,2,3,4]. Microneedles 50–800 μm in length therefore penetrate the stratum corneum and viable epidermis but stop short of pain-sensing nerve endings, opening transient micro-conduits through which drug molecules diffuse into the capillary-rich dermal layer for systemic absorption [3,11,12]. Figure 1 schematically illustrates this mechanism and contrasts the five principal microneedle architectures—solid, coated, hollow, dissolving, and hydrogel-forming (swelling)—that operationalize the four canonical delivery modes (‘poke-and-patch’, ‘coat-and-poke’, ‘poke-and-flow’ and ‘poke-and-release’).

Relative to conventional drug-delivery routes, therapeutic microneedles offer four compounding advantages that are now extensively documented in the transdermal-delivery literature [1,2,3,4,11,12,21,22,23,24,25]. First, by avoiding dermal innervation, they eliminate the pain, needle phobia and bruising associated with hypodermic injection, which directly translates into higher patient compliance—a documented concern for chronic regimens such as insulin therapy, pediatric vaccination and HRT [4,5,6,7]. Second, they bypass the gastrointestinal first-pass metabolism that compromises the bioavailability of orally administered peptides, proteins and many small molecules, while simultaneously circumventing the stratum corneum barrier that limits passive transdermal patches to a narrow window of low-molecular-weight, lipophilic drugs [1,2,3,4]. Third, the self-administered patch format reduces the need for trained personnel and sharps disposal infrastructure, an attribute particularly valued in mass-vaccination campaigns and resource-limited settings [5,23]. Fourth, microneedles enable spatiotemporal control overdosing through choice of architecture: dissolving and hydrogel-forming systems support sustained or stimuli-responsive release; coated devices deliver bolus doses; and hollow microneedles allow active, micropump-coupled infusion of liquid therapeutics [16,17,18,19,20]. Collectively, these attributes make microneedles a clinically attractive alternative to subcutaneous injection, oral tablets and topical formulations whenever painless, dose-accurate and self-administrable delivery is required.

Traditional methods for selecting microneedle research materials are primarily based on trial-and-error, experience, and intuition [25]. However, these methods are not practical for ranking the relative importance of different performance criteria, are not transparent, and do not provide consistent results across different research groups and applications [26]. Moreover, with emerging and current applications for microneedle therapy using a wide variety of materials, conventional selection becomes inefficient in the systematic optimization of this variety [27]. This research work is therefore focused on developing a systematic framework for materials selection for microneedles used in therapeutic applications. In addition to trial-and-error, it optimizes a multi-criteria decision-making process. The approach used in this research is the AHP method for comparing four main types of materials (silicon, ceramics, metals, and polymers) under five significant performance criteria (biocompatibility, mechanical property, cost, ease of manufacturing, and type). The goal is to provide information-based input to determine the materials most likely to be appropriate for the desired application.

### 1.2. Research Contribution and Novelty

This study advances microneedle material selection through three distinct contributions:

Methodological Contribution: The study developed a hybrid decision framework integrating quantitative literature data with expert judgment systematically. Unlike subjective expert panels or purely data-driven approaches, the protocol (Section 2.4.2) translates objective performance metrics into pairwise comparisons, then allows expert refinement based on application-specific considerations. This addresses the “expert black box” critique common in AHP studies.

Scientific Contribution: The study quantified performance trade-offs across five criteria, revealing that biocompatibility dominates therapeutic microneedle selection (48.8% weight), followed by mechanical properties (25.3%). This hierarchy differs markedly from cosmetic microneedles (where cost/aesthetics dominate) and diagnostic devices (where mechanical precision dominates). The 48.8% biocompatibility weight reflects therapeutic applications’ stringent safety requirements under FDA/ISO frameworks.

Practical Contribution: The study provided application-specific decision guidelines mapping material-to-type combinations: polymers for dissolving/controlled-release systems, metals for hollow liquid-delivery devices, ceramics for chemically demanding environments, and silicon for research prototyping. These actionable recommendations address the practitioner need identified in recent surveys [28,29].

Unlike previous reviews [25,26,27] that descriptively compare materials, the study framework provides quantitative priority scores, validated rankings, and sensitivity-tested recommendations for material selection under real-world uncertainty.

## 2. Methodology

### 2.1. Analytic Hierarchy Process (AHP) Model

AHP was selected for its hierarchical, multi-criteria evaluation method, systematic pairwise comparisons, and consistency-checking features [30,31,32]. The methodology has been successfully applied in biomedical material selection [33], where the decision is structured into three levels: goal (choice of the optimal material), criteria (performance indicators), and alternatives (material classes). The silicon MN can be used to make precise geometries using photolithography and deep reactive ion etching, with 150–200 μm penetration depths that are capable of therapeutic delivery [34]. However, brittleness-induced fractures during insertion [35] require insertion forces of 0.1–0.3 N, which can lead to patient discomfort [36]. For ceramic, alumina-based microneedles have shown excellent biocompatibility with structural stability and little inflammatory response [37,38]. High-temperature processing (>1000 °C) and the need for specialized processing equipment are also limitations to manufacturing [39], but chemical inertness is a potential advantage for long-term implantable therapeutic systems [40]. Moving to metallic materials, stainless steel is used for effective hollow insulin delivery [41,42], whereas titanium alloys are biocompatible and have low cytotoxicity [43]. Biodegradable magnesium alloys dissolve; however, their corrosion resistance remains an issue for long-term therapeutic applications [44,45]. Finally, polymeric materials are the most versatile option using PVA and PVP for dissolving microneedles with tunable drug release rate [46], 3D printing capability for insulin delivery with an efficacy similar to that of conventional injection [47], direct inclusion of therapeutic agents in the matrix [48] and pH-mediated controlled release systems [49].

### 2.2. Materials Alternatives

Based on the literature review, four main types of microneedle materials have been identified and summarized in Table 1 as the most widely studied materials for therapeutic applications [50,51]. These materials differ in molecular structure, production, and performance. Polymeric materials, such as PVA, PVP, PCL, PLGA, PLA, and chitosan, offer great versatility due to their design flexibility, which determines biodegradation, mechanical properties, and drug loading. Polymeric materials are used as biodegradable, sustained drug-delivery vehicles that enable microneedle biodegradability and dissolution. They are compatible with various fabrication techniques, including micro-molding and 3D printing, and are suitable for vaccines and proteins. Metal materials (mainly stainless steel, titanium alloy and biodegradable metals such as magnesium and iron) have high strength (yield 200–1000 MPa, modulus of elasticity 100–200 GPa), so that the skin can be stably penetrated and the fluid can be delivered, making them suitable for insulin and electrotherapy. However, as non-biodegradable materials, these metal microneedles raise concerns about sterilization and reusability. Alumina, zirconia, hydroxyapatite, and silica are chemically inert ceramic materials that have high compressive strengths (1000–2000 MPa), biocompatibility, and low tissue trauma when well-designed. They are produced at elevated temperatures via sintering and can thus only incorporate stable small-molecule drugs. Silicon microneedles have been prepared from semiconductor materials, including silicon. However, they can be produced only by photolithography and etching, are brittle and expensive, and achieve penetration depths of only ~150–200 μm, limiting their research applications.

### 2.3. Evaluation Criteria

Evaluation criteria are the most essential features to be established for systematic material selection in therapeutic microneedle applications. After a literature review and consultation with experts in materials science, biomedical engineering, regulatory, and clinical fields, five fundamental criteria were identified [28,29] (Table 2). These are designed to overcome application problems characteristic of therapeutic drug delivery and are therefore distinct from diagnostic or cosmetic applications. They include technical, biological, economic, and practical considerations that impact clinical viability and commercial success, and are prioritized accordingly.

Biocompatibility (C1) is the most critical criterion in selecting therapeutic microneedle materials, as it determines their ability to perform their intended function and elicit an appropriate host response without causing adverse effects. More than non-toxicity, it encompasses other aspects, such as biological interactions at the material-tissue interface during delivery. Biocompatibility affects efficacy and patient safety [52]. Assessment should be based on acute, subacute, and chronic responses, ensuring that cytotoxicity is minimal, sensitization does not occur, and systemic toxicity is low. In addition, it involves assessing interactions with therapeutic agents, patient variability (age, immune status, medications, conditions), and compliance standards (e.g., ISO 10993). In many cases, testing is needed for specific delivery scenarios.

Mechanical Properties (C2) assess the structural integrity, deformation, and failure of the material under complex loading for drug delivery. Microneedles should be hard enough to penetrate the stratum corneum (which has mechanical properties comparable to low-density polyethylene (LDPE), exhibiting an elastic modulus of 100–200 MPa, hardness of 0.4–1.0 GPa (Shore D scale), and ultimate tensile strength of 15–20 MPa [53,54]). This mechanical barrier requires penetration forces of 0.1–0.5 N per microneedle, depending on needle geometry and insertion velocity [55]. Microneedles need to withstand buckling, particularly when used in high-aspect-ratio designs. Their elastic modulus and geometry must be designed to account for this. Fracture resistance is essential for preventing safety problems caused by breakage, particularly in brittle materials such as silicon. Hollow microneedles should be able to sustain internal pressure and have sufficient hoop strength. Fatigue resistance is required for microneedles to remain durable through multiple cycles, which is essential for reusable microneedles. Manufacturing materials also need to withstand processing stresses. Dissolvable microneedles must be capable of penetrating and dissolving at the same time. Performance will be impacted by the temperature during manufacture and storage.

The cost of materials (C3) is another major economic element that determines the practicability and availability of microneedle systems in healthcare practices. This cost includes procurement, processing, quality control, and waste management. Since microneedles are mostly single-use instruments, the need to preserve necessary qualities and avoid cross-contamination are key to popularization, and cost is the key factor in this matter. The costs of raw materials include the low cost of polymers and the high purity of silicon, although overall costs span the whole value chain. The costs of processing can be greater than the costs of raw materials, especially in specialized manufacturing processes like cleanroom operations or high-temperature furnaces. Even tooling needs will vary; silicon production will require expensive photolithography and etching devices, and polymer-based production will use less expensive molding or 3D printing devices. Furthermore, the costs of quality control are higher due to the extensive testing necessary to obtain regulatory approval. Yield rates affect overall costs: high defect rates increase per-unit costs. Different materials require different disposal methods and incur various costs, some of which are environmentally costly. Economies of scale can bring down the cost at high volume for certain technologies but not others. In resource-limited settings or mass vaccination, the material choice may be determined by cost rather than technical benefits.

Ease of Manufacturing (C4) assesses the practicability, technical complexity, and reproducibility of manufacturing therapeutic microneedles at clinical and commercial scales. It takes into consideration process complexity, equipment requirements, environmental controls, scalability, and quality assurance. Easier processes decrease defects, reduce production time, and need less training. Silicon microneedles are much more complex than polymer microneedles, involving processes such as photolithography, whereas polymer microneedles are simpler, such as micro-molding or 3D printing technology. Equipment ranges from expensive semiconductor equipment to less expensive molding systems. For example, silicon requires a restrictive cleanroom environment, while polymers are tolerant of more permissive environments. Scalability involves increasing volume and quality/cost efficiency. Reproducibility means batches are consistently of high quality, and it is crucial for regulatory compliance and market success. For therapeutic use, processes should also include the loading of drugs without damaging the microneedles or the payload.

While the AHP methodology is ideally independent of the criteria, some degree of interdependence is observed in practical applications, such as material selection. For example, the ease of manufacturing (C4) of a material can affect the material cost (C3), and microneedle type compatibility (C5) is intrinsically related to the mechanical properties (C2) and biocompatibility (C1). For this reason, the criteria were specifically formulated to represent a particular view in the decision-making process: biological safety (C1), technical performance (C2), economic aspects (C3), practicality in production (C4), and design variability (C5). This framework must imply that even though there may be underlying factors, each criterion provides a clear and essential context for consideration and prevents significant double-counting in the expert judgment process.

Microneedle Type Compatibility (C5) refers to the compatibility of materials used in different types of microneedles, in recognition of the fact that different therapeutic uses require different configurations and different material needs. Five types exist: solid microneedles for pore formation; microneedles coated with therapeutic agents; hollow microneedles with liquid drugs; dissolving microneedles containing biodegradable drugs; and swelling microneedles comprising hydrogel structures [54]. Solid microneedles require hard materials (metals, silicon); coated microneedles require surface functionalization for drug loading; hollow microneedles require biocompatible polymers with stable channel-forming capabilities; and swelling microneedles require hydrogels for drug delivery. The range of materials that can be used is also limited by the interactions with the drug, stability, and manufacturing requirements for therapeutic applications. For example, an antigen must be delivered in vaccines and be compatible with adjuvants. Delivery time is another factor that affects material choice: The fastest delivery times might be best served by materials that dissolve quickly. At the same time, sustained release requires materials that enable longer delivery. The use of multi-material microneedles provides design and fabrication flexibility for a range of microneedle types.

**Table 2:** Evaluation criteria for therapeutic microneedle applications.

Symbol	Criteria	Description	Importance	Therapeutic Relevance
C1	Biocompatibility	The material’s ability to perform with an appropriate host response in specific therapeutic applications	High	Critical for patient safety and regulatory approval [54]
C2	Mechanical Properties	The material’s ability to maintain structural integrity during insertion and drug delivery	High	Essential for reliable penetration and therapeutic efficacy [14]
C3	Material Cost	The economic considerations for material procurement and processing of therapeutic applications	Medium	Important for commercial viability and healthcare accessibility [23]
C4	Ease of Manufacturing	The complexity and feasibility of fabricating therapeutic microneedle systems	Medium	Critical for scalable production and quality consistency [55]
C5	Microneedle Type Compatibility	The suitability of materials for different microneedle designs and therapeutic delivery modes	Medium	Determines applicability across various therapeutic applications [56]

#### Criteria Selection Process and Exclusions

The five evaluation criteria were selected through a systematic three-phase process. Phase 1 identified 12 potential criteria through a literature review (n = 157 papers, 2015–2024) and expert brainstorming. Phase 2 employed a criteria screening workshop (n = 7 experts) using independence, measurability, and discriminatory power as selection filters. Phase 3 finalized five criteria through Delphi consensus (two rounds, agreement threshold: 85%) (Table 3).

Seven criteria were deliberately excluded with documented justification:

- Drug loading capacity: Subsumed within C5 (type compatibility). Drug loading is type-specific (dissolving MN: integrated; coated MN: surface-applied; hollow MN: post-fabrication) rather than material-inherent.
- Dissolution rate: Covered within C1 (biocompatibility evaluation). Dissolution testing is part of ISO 10993-13 degradation assessment, already factored into biocompatibility scoring.
- Sterilization compatibility: Integrated into C4 (manufacturing). Sterilization (gamma, EtO, autoclave) is a manufacturing step. Material compatibility is established during process validation.
- Environmental impact: Not primary for therapeutic devices. FDA/ISO frameworks prioritize patient safety and efficacy over environmental considerations for single-use medical devices.
- Aesthetic appearance: Non-discriminating for therapeutic use. Unlike cosmetic microneedles, therapeutic devices are not visually inspected by patients during application.
- Patient preference: Non-discriminating across materials. All microneedle types provide painless administration regardless of material. Patient preference relates to application method, not material composition.
- Shelf life: Addressed within biocompatibility (C1) through stability testing per ICH Q1A(R2) guidelines. Material degradation over shelf life is captured in accelerated aging protocols.

Comprehensive coverage of clinically relevant performance dimensions. The five-criterion set (C1–C5) was designed so that every parameter recognized as clinically critical for therapeutic microneedles maps onto at least one of the selected criteria. Drug-material interactions, in particular, are not a single isolable property but a distributed phenomenon that manifests across three of the five criteria: chemical compatibility, immunogenicity, leachables/extractables and degradation-product toxicity all fall under biocompatibility (C1) and are formally interrogated by the ISO 10993 test battery. Structural compatibility of the matrix with the loaded therapeutic (plasticization, embrittlement, drug-induced changes in mechanical integrity) is captured by C2. Architecture-specific drug-loading and release modes (matrix integration in dissolving systems, surface coating efficiency in coated systems, lumen patency in hollow systems, swelling-controlled release in hydrogels) are encoded within C5 (Type compatibility). Penetration mechanics (insertion force, tip sharpness, fracture risk during deployment) are quantified within C2, while sterilization compatibility and scalability are embedded within C4. Cost of goods, reimbursement-relevant economics and supply-chain accessibility are handled within C3. Table 3 documents not only which criteria were selected, but also where each excluded sub-property is captured within the retained set, ensuring complete clinical coverage without double-counting.

Justification for excluding dissolution rate and drug loading as independent criteria. Both parameters were considered explicitly and ultimately rejected on principled grounds of non-independence rather than irrelevance. Dissolution rate is intrinsically a material-degradation phenomenon and is already quantified within the ISO 10993-13 degradation-assessment limb of biocompatibility (C1). Moreover, for the dominant microneedle architectures, the clinically meaningful dissolution profile depends jointly on polymer chemistry (C1), cross-link density and molecular weight (C2), and the specific dissolving/swelling architecture chosen (C5). Treating it as an independent sixth criterion would therefore induce strong cross-correlations with C1, C2 and C5 and inflate the consistency ratio without contributing new discriminative information. Drug-loading capacity is similarly architecture-dependent rather than material-inherent: the same biocompatible polymer can be matrix-loaded (dissolving), surface-coated, or used as a hydrogel reservoir, with loading capacities differing by an order of magnitude between modes. Loading is therefore subsumed within type compatibility (C5), where the polymer-matrix loading windows (10–40 wt% small molecule; 1–10 wt% protein), coated-surface loading (0.1–10 μg/needle) and hollow-reservoir volumes (≥0.1 μL) are explicitly weighted. Sensitivity analyses (Section 3.3.3) confirm that the polymer–metal top-two ranking is preserved when dissolution-related sub-scores are perturbed by ±20%, supporting the conclusion that explicit promotion of these sub-criteria would not alter the practical selection guidance.

### 2.4. Pairwise Comparison Process

The basic scale (Table 4) is used in the AHP methodology to compare relative preferences between criteria and therapeutic microneedle alternatives through pairwise comparisons [55]. This systematic tool allowed specialists to make multi-criteria decisions more complex and consistent by comparing the materials and criteria in pairs.

**Expert Judgment Process of Collection and Comparison**

Expert judgments were obtained through structured interviews in which knowledge of the materials science, biomedical engineering, and regulatory affairs disciplines [56] was elicited. The experts systematically compared pairs of materials and criteria using the nine-point scale shown in Table 3. For instance, in the case of biocompatibility versus mechanical properties, the researchers needed to know which was more critical for therapeutic microneedles and how important each one was. Similarly, when comparing polymers against metals in terms of biocompatibility, experts determined which material performed better and assigned an appropriate intensity value.

The comparison process resulted in several matrices: a 5 × 5 matrix comparing the five evaluation criteria, and five individual 4 × 4 matrices comparing the four materials for each criterion. The relative preference of one element over another, or the relative performance of one aspect over another element, was denoted in each of the matrix entries. The diagonal elements of these matrices were necessarily identical because all aspects compared with themselves are identical, and the lower part of each of the matrices was filled with reciprocal values to provide mathematical consistency.

**Significant Pairwise Comparisons Findings**

The pairwise comparison process identified different patterns of performance between materials and criteria. For the biocompatibility comparisons, in particular, polymers received strong-to-very-strong preference ratings (scale values 5–7) from the experts, compared to the other materials. This was due to abundant literature on the use of polymers in pharmaceutical applications, their low inflammatory response, and their biodegradable properties. Silicon had the lowest biocompatibility, and metals had moderate ratings; there have been reports of cytotoxicity and surface treatment problems.

Mechanical properties were the other way around, in which metals ranked the best among other materials. The preference ratings assigned to metals (scale values of 5–7) were higher than those for polymers because of their relative advantages in tensile strength, fracture resistance, and the ability to maintain structural integrity during skin penetration. Silicon and ceramics were given a moderate rating in brittleness, while polymers were given the lowest rating for mechanical performance, but were acceptable for single-use dissolving applications.

Polymers again provided the apparent advantage of ease of manufacture. Comparisons were made with regard to compatibility with the existing pharmaceutical production infrastructure (molding, casting and 3D printing processes). Experts gave higher preference values to polymers (scale value 5) than to metals, and very high preference values to ceramics and silicon (scale value 7), which require special clean-room facilities and high-temperature processing, thereby introducing significant complexity to the manufacturing process.

The type-compatibility tests showed a stronger relationship between performance and microneedle type. The outcomes for both metals and polymers were favorable, and both were suggested for use in other instances. Polymers were preferred for dissolving and swelling microneedles (strong preference, scale value 5), but metals were chosen for hollow microneedle applications, where the accuracy of fluid-delivery channels was paramount. This is application-specific, and thus it represented fair performance as the best choice of material would depend on the goal of any therapeutic use.

Type compatibility proved to be the most architecture-dependent of the five criteria. Polymers and metals performed well overall but in different niche applications: polymers were preferred for dissolving and swelling microneedles (preference 5), while metals were preferred in hollow architectures where the channel architecture needs to be tightly controlled. In this case, it is not only the material’s intrinsic characteristics that determine the best material but also its therapeutic use.

**Calculation of Priority Vectors and Results**

The mathematical algorithm includes calculating the priority vectors via eigenvalue decomposition and analyzing the consistency ratio [55]. The priority weights were computed using the eigenvalue method based on matrices of pairwise comparisons. The eigenvector was obtained and normalized to yield priority weights that sum to 1 for each matrix. Local priorities are the comparative scores of significance or performance ranking of each component within its comparison group.

For material performance based on a single criterion, the normalized local priorities reflected the merits of comparison, as revealed by pairwise comparisons. Polymers had a local priority of 0.530 for biocompatibility, which was significantly higher than metals (0.212), ceramics (0.212), and silicon (0.046). On the other hand, metals prevailed (0.503) in local priority compared to polymers (0.035) only. For the ease of manufacture, polymers had a local priority of 0.533, and for cost, this priority was 0.622, which are clear advantages in ease of manufacture and cost rationality.

The local priorities show the same patterns that can be seen in the pairwise comparisons within each criterion. Polymers outperform metals and ceramics, which are both ahead of silicon (0.046) and level with the former at 0.212, while biocompatibility scores are at 0.530 on average. Mechanical properties are dominated by metals (0.503), and reduced in polymers to 0.035 in this dimension. However, the advantage of polymers in ease of manufacturing (0.533) and cost (0.622) is clearly shown to be the lead.

**Consistency Verification**

The consistency ratio (CR) was calculated using Saaty’s standard Random Index for a 5 × 5 matrix (RI = 1.12). The maximum eigenvalue (λmax = 5.091) yielded a consistency index (CI) = (5.091 − 5)/(5 − 1) = 0.023. Thus, CR = 0.023/1.12 = 0.020 (2.0%). This is well below the 0.10 threshold, indicating excellent consistency in expert judgments. For all the criteria, the value of principal eigenvalue lm was obtained from the criteria comparison matrix, and the consistency index (CI) = (lm − n)/(n − 1) was calculated, where n = 5, i.e., the number of criteria. This consistency index was then divided by the random index (RI = 1.12 for a 5 × 5 matrix) to get the consistency ratio. The resulting CR = 0.020 (2.0%) was well within the acceptable range (CR < 0.10), suggesting that experts’ judgments were logically consistent and free of essential contradictions.

The pairwise comparisons were carried out systematically rather than randomly, and the acceptable consistency ratio was used to verify them. The CR value below the 0.10 threshold indicated that the experts had reasonable transitivity in their judgments; that is, if polymers were judged superior to metals, and metals were judged to be superior to ceramics, then polymers were considered to be superior to ceramics by an amount that was appropriate for these relations. Similar consistency checks were also performed for each material comparison matrix, all of which yielded acceptable consistency ratios and therefore are consistent with the derived priority weights.

**Global Priority Synthesis**

The local priorities in the individual comparison matrices were combined by using weighted summation to produce global rankings. The global priority of each material was calculated as Pi = summation (wj × pij), where wj is the weight of criterion j and pij is the local priority of material i with respect to criterion j. The multivariate synthesis technique, based on the criteria’s importance weights and material performance values, was applied to generate a general preference matrix.

When all five criteria are folded together, it gives the balance of strengths and weaknesses of each material. Biocompatibility is the key factor in the polymer ranking, followed by polymer advantages in manufacturing and cost (0.530 × 0.489 = 0.259). Polymers do not do very well on mechanical properties, but their moderate weight on that criterion (0.253) restricts the damage. The biocompatibility and manufacturability scores are lower than that of metals, which more than make up for the large mechanical contribution (0.503 × 0.253 = 0.127). The synthesis is thus automatic, with the importance of each criterion for therapeutic use determining its weightage.

Pairwise comparison and mathematical synthesis resulted in the following ranking of expert knowledge into quantitative priorities: polymers (0.383 of the global priority), metals (0.318 of the global priority), ceramics (0.176 of the global priority), and silicon (0.123 of the global priority). These rankings were derived from aggregated expert opinion on the therapeutic suitability of microneedles, considering all 5 evaluation criteria together and weighting them according to their relative importance for achieving safe, effective, and practical drug-delivery systems.

#### 2.4.1. Expert Panel Selection and Judgment Collection

A multidisciplinary expert panel of seven specialists was recruited through purposive sampling targeting professionals with minimum 5 years of experience in microneedle development, biomaterials, medical device design, or regulatory affairs (Table 5). Expert judgments were collected through individual structured interviews lasting 20–60 min each, conducted between January and March 2025. Each expert independently completed pairwise comparison matrices for all five criteria and four alternatives across each criterion. Individual consistency was verified for each expert’s comparison matrix. All experts achieved acceptable consistency ratios (CR < 0.10), with individual CRs ranging from 0.012 to 0.087. Individual expert matrices were aggregated using the geometric mean method: GM_ij_ = (∏k = 1 → 7 a_ijk_)^1/7^, where aijk represents the pairwise comparison value from expert k comparing alternatives i and j. Inter-rater reliability was assessed using Kendall’s coefficient of concordance (W = 0.82, *p* < 0.001), indicating strong agreement among experts despite their diverse backgrounds.

Mitigation of expert subjectivity and cognitive bias. Although pairwise comparison inevitably relies on expert judgement, five complementary procedures were implemented specifically to attenuate the bias known to affect AHP elicitation. (i) Anchoring bias was mitigated by randomizing the order in which criterion and alternative pairs were presented to each expert and by withholding any pre-existing weights or rankings from the elicitation interface. (ii) Framing and halo effects were reduced by presenting each pair in isolation, with criterion descriptions reformulated in performance-neutral language and accompanied by quantitative reference data from the literature review. (iii) Disciplinary bias was addressed through purposive stratification of the panel across four disciplines (materials science, biomedical engineering, regulatory affairs and clinical practice), ensuring no single perspective dominated the geometric-mean aggregation. (iv) Inconsistent or extreme judgements were flagged in real time using the embedded consistency-ratio calculator; experts with CR > 0.10 on any sub-matrix were asked to revisit the contributing comparisons before submission, a procedure analogous to a Delphi feedback round. (v) Group-level stability was verified post hoc through Kendall’s W (0.82, *p* < 0.001), the absence of outlier matrices on Mahalanobis-distance screening, and Monte Carlo perturbation of weights (±20%) which preserved the top-two ranking in 96.8% of 10 000 simulations. Together, these procedures do not eliminate subjectivity—a residual feature of any judgement-based MCDM method—but they bound its influence and make it auditable.

#### 2.4.2. Literature Data Integration and Pairwise Comparison Protocol

The pairwise comparison process integrated both quantitative literature data and expert judgment through a systematic four-step protocol (Table 6):

Step 1: Literature Data Compilation: A comprehensive literature review extracted quantitative performance metrics for each material across all five criteria. Sources included peer-reviewed publications (2015–2024), ISO/ASTM standards, and regulatory documents.

Step 2: Performance Categorization: Quantitative metrics were translated into performance categories using standardized thresholds:

- Excellent: Performance in the top 25% of reported values
- Good: Performance in the 25–50% range
- Fair: Performance in the 50–75% range
- Poor: Performance in the bottom 25% of reported values

Step 3: Initial Saaty Scale Translation: Performance category differences were mapped to Saaty’s 1–9 scale:

- Same category → 1 (Equal importance)
- One category difference → 3 (Moderate importance)
- Two category differences → 5 (Strong importance)
- Three category differences → 7 (Very strong importance)
- Four category differences → 9 (Extreme importance)

Step 4: Expert Refinement: Initial scores were presented to experts with supporting literature. Experts could adjust scores by ±1 point on the Saaty scale based on application-specific considerations or recent developments not captured in the literature. Adjustments required written justification.

### 2.5. Criteria Weight Determination

Criteria weights (Table 7 and Table 8) were determined through systematic pairwise comparisons informed by expert knowledge from the materials science, biomedical engineering, and regulatory affairs domains [56].

Using the pairwise comparison matrix, the study obtained different prioritization patterns for the evaluation criteria. Biocompatibility (C1) was considered overwhelmingly dominant in expert assessments, being strongly preferred (intensity 9) over material cost (C3), quite strongly preferred (intensity 7) over ease of manufacturing (C4), moderately to strongly preferred (intensity 5) over microneedle type compatibility (C5) and moderately preferred (intensity 3) over mechanical properties (C2). The preference hierarchy created shows limitations in the interactions between tissues and devices that are caused by the needs of transdermal delivery.

The distribution of weights is concentrated in the upper tier, and 48.9 percent of the total importance is represented by biocompatibility. Combining biocompatibility with mechanical properties accounts for 74.2% of the decision priority. This huge allocation is given the importance of such biological safety and structural integrity in the choice of biomaterials to use as microneedles. The stratified importance ranking is also emphasized by the wide gap between the most important criterion (C1: 0.489) and the least important one (C3: 0.035)—almost 14-fold.

Mechanical properties (C2) were the second, with a weight of 25.3, which is about half that of biocompatibility. This ranking is in agreement with the expert opinion that although mechanical performance is key to efficient skin penetration and structural stability, it is not primary in comparison to safety considerations. The pairwise comparisons indicate C2 to be a strong choice (intensity 7) compared to material cost, moderately to strongly favored (intensity 5) compared to ease of manufacturing, and moderately favored (intensity 3) compared to microneedle type compatibility.

Microneedle type compatibility (C5) is in the middle tier, accounting for 15.8% of the biocompatibility weight and almost two-thirds of the mechanical properties weight. The comparisons show that this criterion was moderately to highly preferred (intensity 5) over both material cost and ease of manufacturing but was significantly lower than the first two criteria. This middle-ground position suggests that although the ability to produce microneedle devices with multiple geometries is desirable, it remains clearly secondary to performance and safety.

The combined weight of C3 (Material Cost, 6.6%) and C4 (Ease of Manufacturing, 15.8%), totaling 22.4%, reflects therapeutic microneedles’ regulatory and clinical priorities. This weighting distribution is deliberately lower than economic criteria because:

**Clinical Safety Supersedes Cost:** Under FDA/ISO frameworks, therapeutic devices prioritize patient safety (biocompatibility) and performance (mechanical properties) over manufacturing economics. FDA guidance explicitly states: “The benefits of the device must outweigh its risks” [21 CFR 860.7], with no cost consideration in benefit–risk calculus. Expert panel consensus (Kendall’s W = 0.82, *p* < 0.001) reflected this hierarchy, with clinical experts (E5, E6) uniformly rating safety 7–9× more important than cost.

**Commercial Viability Remains Above Threshold:** While weighted lower than clinical criteria, the 22.4% combined manufacturing/cost weight ensures commercially non-viable materials are eliminated. For reference, cosmetic microneedles show inverted priorities (cost/manufacturing: 55–60%, biocompatibility: 20–25%) [57], whereas diagnostic devices emphasize precision/cost (mechanical: 50%, cost: 25%) [58].

**Sensitivity Analysis Confirms Appropriateness:** Monte Carlo simulations (10,000 iterations) tested alternative weight distributions. Increasing C3 + C4 to 35% did not change the top two rankings (polymers, metals), while decreasing to 10% maintained identical rankings but reduced discriminatory power for positions 3–4.

**Economic Realities Embedded in Other Criteria:** Manufacturing cost influences biocompatibility assessment (expensive validation studies favor simpler materials) and type compatibility (manufacturing complexity constrains design options). Thus, economic considerations appear indirectly beyond the explicit C3/C4 weights.

The consistency ratio (CR = 0.020), which falls within the acceptable range (CR < 0.10) for group decision-making, was used to assess consistency [55]. The CR value indicated that expert judgments were reasonable overall in the pairwise comparison procedure, and the deviations from perfect consistency were within acceptable statistical levels. The ratio indicates a relative lack of similarity in the comparison matrix of about 2.0%, which could be attributed to the difficulty of simultaneously evaluating five elements across several dimensions. The comparison can be subjective because the expert panel is interdisciplinary and because the qualitatively different characteristics of biocompatibility and subjectivity should be considered. Nevertheless, the consistency which has been observed shows that there is a great level of agreement among the experts. The validation procedure ensures that the weights set reflect the overall verdict of the specialists, as opposed to being affected by personal influences or opposing views.

Further information is derived from the relative positioning between the manufacturing and cost criteria. Although material cost was rated slightly more preferred (intensity 3) than ease of manufacturing in direct comparison, manufacturing ultimately received a higher final weight (6.6% vs. 3.5%), as shown above. This apparent reversal is due to the relative performance of ease of manufacturing being much better than material cost, compared with microneedle type compatibility (both are equally preferred and show a slight preference for ease of manufacturing), and material cost being slightly worse than ease of manufacturing (they are equally preferred). For this reason, the sum of all the pairwise ties raised manufacturing from cost to first place in the final ranking.

### 2.6. Complete Pairwise Comparison Matrices and Eigenvector Calculations

This section presents all pairwise comparison matrices used in the AHP analysis, including the criteria comparison matrix and material comparison matrices for each criterion. Each table shows the complete matrix, derived eigenvector (local priorities), maximum eigenvalue (λmax), consistency index (CI), and consistency ratio (CR) (Table 9, Table 10, Table 11, Table 12 and Table 13).

### 2.7. Evaluation of Material Property with Literature Verification

The systematic evaluation of materials to be potentially implemented into therapeutic microneedle systems was carried out through an analysis of the literature and a set of standardized testing protocols. The assessment involved a combination of quantitative outcomes of the standardized tests and the qualitative outcomes of the peer-reviewed literature on properties relating to drug delivery systems [59].

#### 2.7.1. Biomedical Compatibility Studies

Materials that are to be used in transdermal treatment are also subject to strict conditions of biocompatibility. The assessment was conducted in accordance with the ISO 10993 series, internationally acclaimed standards for testing medical devices [52]. Cytotoxicity was evaluated as per the ISO 10993-5 guidelines, using L929 mouse fibroblast cells as the reference line in a direct contact cytotoxicity test. This process identifies materials that emit toxic substances that can be damaging to living cells. The cell cultures were incubated with material extracts between 24 and 48 h, and cell viability was determined by performing metabolic activity assays. Polymeric materials exhibited high cell viability, which was over 90 percent, implying low cytotoxicity. The compatibility of silicon-based materials was moderate, and the results depended mostly on the method used to treat the samples, which could be oxidation or the introduction of a coating [57].

The possibility of skin sensitization was measured in two complementary tests: the Guinea Pig Maximization Test and the Local Lymph Node Assay, which was done in compliance with ISO 10993-10. These tests are used to determine if repeated exposure to the same substance causes allergic immune responses. Medical-grade polymers have repeatedly shown low sensitization responses across various studies and are therefore a preferred option for repeated or long-term skin-contact applications [58,60]. Systemic Toxicity: ISO 10993-11 protocols were used to assess potential systemic toxic effects resulting from material implantation or systemic exposure. This included determining if material components or degradation products entering the bloodstream produce adverse effects on the central organ system(s). Polymeric materials exhibited good systemic compatibility profiles, with most of degradation products being considered to be of low toxicity or metabolites [57].

#### 2.7.2. Mechanical Characterization of Properties

Mechanical properties directly affect microneedle performance, including successful insertion into the skin, structural integrity during insertion, and drug-delivery reliability. Comprehensive mechanical testing was done using internationally recognized standardized protocols to ensure comparability and reliability of the data (Table 14).

**Tensile Testing:** The material’s strength and elasticity were characterized according to ASTM D638 for polymers and ASTM E8 for metals and ceramics. Controlled experiments were performed at environmental conditions of 23 ± 2 °C and relative humidity of 50 ± 5% percent with standard specimen geometries. Tensile tests were used to measure how the materials would behave when subjected to tensile loading, which is an important consideration when processing and handling microneedles. The tests provided quantitative data on the elastic modulus, ultimate tensile strength and elongation at break.

**Compression Testing:** A special compression-testing device was designed to apply compressive forces during the insertion of the needle tip. The tests were used to measure the force necessary to puncture the skin successfully, the fracture resistance and the deformation of the microneedle when subjected to a force. The data obtained was directly used to help choose needle materials and treatment modalities.

**Fracture Mechanics:** Stress intensity factors (KIC) that are critical to fractures were calculated using ASTM E399 to determine the material’s resistance to extreme crack propagation. The ability of microneedles to penetrate the skin and form a mark upon usage is a critical feature for forecasting catastrophic modes of failure.

Mechanical property data indicate that silicon and ceramics are highly rigid and possess low fracture toughness; thus, they are brittle and can easily break. Metals are strong as well as fracture-tough and thus are able to bend and break. Polymers are more flexible and have intermediate mechanical properties; hence, a design using polymers can offer flexibility.

#### 2.7.3. Surface Characterization and Compatibility of Drugs

Surface properties control the most important interfacial events, including the adhesion of drug coating, loading capacity, release kinetics, and the biological interaction at the skin interface.

**Surface Analysis:** Atomic force microscopy (AFM) was used to measure surface topography at the nanometer scale, and it was found that material-specific roughness patterns affected coating homogeneity and the strength of adhesion of drugs [66]. Smoother surfaces were found to have more even coatings of drugs, and controlled roughness was used to increase mechanical interlocking in specific coating recipes.

**Surface Chemistry:** X-ray photoelectron spectroscopy (XPS) was used to characterize the chemical composition of surface functional groups. This experiment is critical for identifying how proteins adsorb and for evaluating possible immunological reactions at the skin–device interface. The amount of surface oxidation, the existence of hydroxyl groups, and the carbon content had a strong impact on biological interactions [67].

**Accessibility Wettability:** Contact angle measurements, which are used to measure the angle between a liquid droplet and the material surface, were used to assess surface hydrophilicity and hydrophobicity. These measurements are instant indicators of the strength and uniformity of drug coating, especially when using aqueous-based formulations. Materials with intermediate wettability, with contact angles between 40 and 70 degrees, showed better coating properties [68].

**Drug Compatibility Studies:** The polymeric material was tested for compatibility with therapeutic agents and its ability to regulate drug release. The efficiency of drug incorporation was measured as the percentage of drug incorporated into the polymer matrix. Kinetic experiments were conducted with drug-loaded samples placed under physiological conditions to measure the rate of drug accumulation over time. Zero-order, first-order, Higuchi and Korsmeyer–Peppas equations were used to model the release mechanisms, with the aim of differentiating between diffusion-controlled, dissolution-controlled and combined processes [49]. Tests were carried out to predict shelf life and guarantee therapeutic efficacy and stability over time, and accelerated aging tests were performed to comply with ICH Q1A guidelines [69].

#### 2.7.4. Process of Evaluation of Manufacturing

Ease of manufacturability is a crucial factor in the selection of materials, particularly with respect to scaling, reproducibility and economic feasibility in therapeutic practices. Additive Manufacturing Potential: Stereolithography (SLA) and other photopolymerization methods can produce polymer microneedles (25–50 µm feature resolution) that are sufficient to meet most therapeutic requirements [47]. This technology allows for the creation of complex geometries that are impossible to achieve using conventional manufacturing methods. Processing Requirements: The processing requirements for different classes of materials vary in complexity and capital investment. Silicon microneedles also require semiconductor manufacturing technology, such as cleanroom facilities, photolithography, and etching technology [34]. Metal microneedles are machined to a high level of precision, electroformed, or stamped with high precision, requiring high tolerance and special tools [41]. The sintering of ceramic materials is done at high temperatures (>1400 °C), and the heating and cooling cycles must be strictly controlled so that no cracking of the materials occurs [37]. In contrast, polymeric materials have high flexibility in processing choices, including injection molding, hot embossing, micro-molding, and other 3D printing techniques, with relatively fewer requirements for processing equipment [53].

The cost analysis (Table 15) shows that although polymeric materials may offer lower costs of raw materials and less demanding processing methods, a significant cost disparity would be experienced in therapeutic applications due to the requirements of addressing and upholding medical-grade material specifications and quality assurance measures. In the sphere of commercial therapeutic manufacturing, polymers are the least expensive, and silicon is the most expensive, with regard to its specialized facility needs.

#### 2.7.5. Statistical Analysis and Validation

The material assessment framework was to be checked properly to guarantee the reliability of the framework and make the decision-making processes confident (Table 16).

**Reliability Assessment:** The reliability of expert judgements and decision models was assessed using a variety of techniques. The consistency of expert assessments was confirmed by repeated assessments, and the stability of the overall decision scheme was challenged by fluctuating input parameters within reasonable ranges to see if material rankings remain stable.

**Experimental Design:** The study used a factorial-type of Design of Experiments (DOE); this allowed measuring multiple material properties concurrently, performing fewer experiments. The approach would be able to determine the key effects and interactions among various material properties, giving a complete picture of how the various material properties relate with each other.

**Validation Framework:** The AHP methodology includes built-in consistency-checking mechanisms to detect and correct inconsistent pairwise comparisons. Additionally, other MCDM approaches, such as TOPSIS and weighted sum methods, were also applied to the data, and the rankings of the materials were checked to ensure that the results were consistent across the different analytical frameworks.

The validation framework shows that the silicon, metal, and polymer assessments have an extensive literature base and available databases and, thus, high confidence in the characterization of their properties. Ceramic materials had a moderate amount of literature but used more expert knowledge and extrapolation from closely related applications, so the confidence level in this assessment was moderate. This approach to validation can ensure transparency of the decision-making process and allow users to consider the reliability of the evaluation when interpreting material selection recommendations.

### 2.8. Consistency Verification

Consistency verification is the process of making sure that the expert judgments and comparisons made during material evaluation are logical and consistent. When experts compare materials using different criteria, there may be contradictions in their assessments. For example, if Material A is judged to be better than Material B, and Material B is better than Material C, then logically Material A should be better than Material C. However, human judgments sometimes fail to satisfy transitivity and are therefore inconsistent. The consistency check procedure examines the entire set of pairwise comparisons to identify possible contradictions. The study analysis yielded a consistency ratio of 0.082, well below the maximum acceptable value of 0.10. This means that the expert evaluations have good internal consistency and are reliable for decision-making. In practical terms, less than 10% of the judgments have logical contradictions if the consistency ratio is less than 0.10. This degree of consistency is considered acceptable in group decision-making settings where some variation in experts’ views is expected. Higher consistency ratios indicate that the evaluation process requires revision and that experts should rethink the comparisons or clarify the criteria being evaluated. Verification of consistency is also used to identify which comparisons are objectionable. In situations where inconsistencies are identified, reviewers can focus on the comparisons that contribute the most to the inconsistency to narrow the review process. This repetitive process will result in improved overall quality and reliability in the decision to select the material.

The sensitivity analysis revealed that the ranking is robust to changes in sensitive parameters. The metals would need to reduce their biocompatibility weight to less than 0.30 to be better than polymers, which is a firm conclusion [70]. This analysis is a study of how the various weights of the multiple criteria change. This evaluation demonstrates the quality of the choice of material. For example, the weight given to biocompatibility is now the highest in the evaluation system. The analysis indicates that despite a reduction in the weight of the biocompatibility imperative (to 0.30), polymeric substances will remain the most suitable choice with respect to the use of microneedles in therapeutic applications. This observation implies that polymeric materials are not selected based on a single parameter but rather in line with high performance across an array of evaluation criteria. This strength will ensure that the recommendation for the material will not shift with reasonable alterations in the priority of the various criteria, based on particular application needs or changes in clinical needs.

## 3. Results

### 3.1. Criteria Weight Derivation and Consistency Analysis

The AHP analysis determined the weights of the criteria by constructing a pairwise comparison matrix on a 9-point scale (1 = equal importance, 9 = extreme importance). Eigenvalue decomposition was used to obtain priority vectors by means of the relative weights. Each of the comparison matrices was then normalized to the principal eigenvector (lmax) [70].

**Mathematical Verification Process:** The global priorities were also calculated using matrix multiplication, ensuring mathematical consistency at every tier of the hierarchy. The consistency ratio (CR) was obtained by the formula CR = CI/RI, where CI = (λmax − n)/(n − 1), and RI is the random index of the matrix order n. The comparison matrices all satisfied the acceptable level of CR below 0.10.

**Expert Consensus Validation:** Inter-rater reliability was determined with the help of Kendall’s coefficient of concordance (W = 0.82), which is more than the acceptable level of 0.7 required to reach consensus in a group decision-making. Expert agreement on all criteria rankings was statistically significant, as supported by the ch 2 test (χ^2^ = 164.3, *p* < 0.001).

**Cross-Validation Inter-method:** The entropy-weight method was also employed as a method of objective validation to obtain weights calculated on the basis of information entropy. It was calculated as wi = (1 − Ei)/Σ(1 − Ej) where Ei is the value of the entropy of criterion i. The rank correlation coefficient (ρ = 0.89, *p* < 0.05), obtained through Spearman’s rank correlation method, showed a high level of agreement between the expert weight obtained through the AHP method and the weight obtained through entropy.

The weights were re-elicited 6 months after the initial session to assess temporal stability. The greatest change in absolute value was a decrease of 0.014 in the biocompatibility weight, which is within the ±0.05 tolerance, considered stable for decision purposes. There was high overall stability of the coefficient of variation, which remained below 0.15 for each criterion (Table 17).

### 3.2. Material Performance Evaluation Against Individual Criteria

The AHP technique was applied to the complete pairwise comparisons of all four material groups with five quantitative factors, producing consistency ratios less than 0.10 and the ability to identify distinct performance patterns through quantifiable technical parameters [59]. The local priority scores of each material, shown below, reflect the contribution of the material (as a percentage) to this particular criterion before being summed together to form the global score.

#### 3.2.1. Biocompatibility Analysis (C1)

The biocompatibility analysis (C1) is intended to assess the biocompatibility of the products to be manufactured and to estimate the rejection rate as a percentage. The biocompatibility evaluation followed ISO 10993 series standards, including quantitative cytotoxicity testing per ISO 10993-5, sensitization testing according to ISO 10993-10, and systemic toxicity testing in line with ISO 10993-11 [52].

**Polymeric Materials:** Shown to be highly biocompatible, with cell viability rates exceeding 95% in L929 fibroblast cultures after 72 h exposure. Polymers such as polyvinyl alcohol (PVA, Mw 89,000–98,000), polyvinylpyrrolidone (PVP K30, Mw~58,000), and biodegradable polyesters (PLGA 50:50, Mw 30,000–60,000) have low or no inflammatory response (IL-6 level < 50 pg/mL, TNF-α level < 25 pg/mL) to macrophages. The modification of functional groups can be used to exercise tight control. Carboxyl-terminated PLGA degrades 40 times faster than ester forms due to its molecular architecture.

**Silicon Materials:** Current variable biocompatibility is related to the surface treatment procedures. Layers 2–3 nm of native oxide (SiO_2_) provide chemical stability, with contact angles of 45–65°. Silicon (H_2_SO_4_:H_2_O_2_ 3:1 at 120 °C) is cleaned to generate hydrophilic surfaces with contact angles less than 10° and silanization with octadecyltrichlorosilane produces hydrophobic surfaces with contact angles greater than 110° [57]. Grades 0 (no cytotoxic effect) of oxidized surfaces to Grade 2 (mild cytotoxicity) of unpassivated silicon are all cytotoxic.

**Metallic Materials:** Known for their biocompatibility due to widespread use in medical implants. Titanium grade 2 (ASTM F67) and Ti-6Al-4V ELI (ASTM F136) demonstrate excellent osseointegration, with bone-implant contact ratios exceeding 80% after 12 weeks. Stainless steel ASTM F138 exhibits a corrosion rate of less than 0.13 mm/year in simulated body fluid (SBF) at 37 °C [58]. Surface roughness parameters (Ra 0.1–0.4 mm) significantly influence protein adsorption, with fibronectin adsorption being three times higher on surfaces with Ra 0.4 mm compared to polished surfaces.

**Ceramic Materials:** Excellent chemical inertness and dissolution rate of SBF less than 0.01 mg/cm^2^/day. Alumina (Al_2_O_3_) exhibits a hardness of 18–20 GPa and chemical stability at pH 4–10. Zirconia (ZrO_2_) has a higher fracture toughness (6–10 MPa.m1/2) and maintains phase stability at body temperature [37].

Normalized Biocompatibility Priorities: polymers (0.530), metals (0.212), ceramics (0.212), silicon (0.046).

#### 3.2.2. Mechanical Properties Analysis (C2)

Standardized testing protocols, tensile testing (ASTM D638), flexural testing (ASTM D790), and nanoindentation according to ISO 14577 were applied to evaluate mechanical properties (at the microscale) [14] (Table 18).

**Metallic Materials:** Metals exhibited high mechanical strengths, with yield strengths between 200 and 1200 MPa and ultimate tensile strengths between 300 and 1400 MPa. The Young’s modulus of titanium alloys is 110–120 GPa, which is quite close to that of cortical bone (15–30 GPa) compared to other materials. Stainless steel 316L has a yield strength of 205–310 MPa and an elongation of 40–50%, with plastic deformation possible upon fracture. The fatigue life exceeds 107 cycles under a stress level of 50% of the yield strength [41,43].

**Silicon Materials:** Silicon exhibits anisotropic mechanical properties, with a Young’s modulus of 130–169 GPa in <100> and <111>, respectively. Single-crystal silicon has theoretical strengths of 12–20 GPA and practical strengths of 150–300 MPa due to surface flaws and stress concentrations. Their fracture toughness is low, 0.7–1.0 MPa·m^(1/2), which leads to catastrophic brittle failure [35,36]. A Weibull modulus of 5–15 indicates a significant variation in strength.

**Ceramic Materials:** They have compressive strength (2000–4000 MPa in the case of alumina) and low tensile strength (200–500 MPa). The Young’s modulus is 200–400 GPa, and the plastic deformation is very low. Fracture toughness is 3–6 Mpaam for alumina and and 6–10 Mpaam 1/2 for zirconia, respectively. Magnitudes of critical flaws under the Griffith criterion range from 10 to 50 μm, requiring defect-free manufacturing [37,39].

**Polymeric Materials:** Demonstrate a viscoelastic behavior and time and temperature-dependent properties. PLGA has a Young’s modulus of 1.4–2.8 GPA and a tensile strength of 41–55 MPa. PVA has a modulus of 0.1–4.0 GPa, which varies as a function of molecular weight and level of hydration. Creep compliance follows a power law behavior, with exponents of 0.1–0.3 for biomedical polymers [46,47].

**Normalized Mechanical Property Priority:** 0.503 for metals, 0.231 for silicon, 0.231 for ceramics, and 0.035 for polymers.

#### 3.2.3. Material Cost Analysis (C3)

The cost analysis includes costs of raw materials, processing costs, quality control needs, and the manufacturing scalability cost analysis through the activity-based costing approach [71] (Table 19 and Table 20).

**Polymeric Materials:** Demonstrate cost advantages, with raw material costs of $2–15/kg for medical-grade polymers. PVA (Mw 89,000) costs $8–12/kg, PLGA 50:50 costs $150–300/kg, and PVP K30 costs $15–25/kg. Processing has used standard pharmaceutical equipment, with injection molding cycle times of 30–120 s and energy consumption of 0.3–0.8 kWh/kg. Quality control is based on ICH Q7 recommendations and employs conventional analytical procedures (GPC, DSC, FTIR), and costs between $50–200 per batch [47,53].

**Metallic Materials:** This needs to be accurate in production. The cost of Titanium grade 2 raw material is $15–25/kg, and 316L stainless steel is $3–8/kg. CNC machining has a rate of 0.1–2.0 mm^3^/s, and the cost of the tool is $50–200 per insert. Passivation treatments (1–3/part) or electropolishing (2–5/part) are required in surface finishing. Dimensional tolerances of ±5–25 μm require CMM inspection costing $100–500 per measurement protocol [41].

**Ceramic Materials:** Involve high-temperature sintering at 1400–1700 °C, with energy consumption of 15–25 kWh/kg. Alumina powder costs $5–15/kg, and zirconia powder $25–50/kg. Sintering shrinkage of 15–20% requires oversized green bodies and post-sintering grinding with diamond tooling ($200–500/grinding wheel). Densification >98% theoretical density requires controlled atmosphere furnaces costing $200 K–500 K [37].

**Silicon Materials:** Require semiconductor-grade facilities with Class 100 cleanroom specifications. Silicon wafer costs $0.50–2.00/cm^2^ for 4–6 inch diameter wafers. Photolithography using i-line (365 nm) steppers costs $500–$1500 per wafer. Reactive ion etching with SF_6_/O_2_ chemistry occurs at 0.1–0.5 μm/min, with equipment costs of $1–3 M. Metrology requirements include SEM inspection ($50–200/measurement) and profilometry ($25–100/measurement) [34].

**Normalized Cost Priorities:** 0.622 for polymers, 0.224 for metals, 0.077 for ceramics, and 0.077 for silicon.

While polymers demonstrate relative cost advantages compared to metals, ceramics, and silicon, the term “low cost” requires important contextualization for therapeutic applications. Medical-grade polymers (PLGA, PLA, PVA) cost $150–300/kg versus $2–15/kg for commodity grades [56]. Additional pharmaceutical manufacturing requirements substantially increase unit economics:

- GMP compliance adds 20–50% overhead depending on production scale
- Sterilization validation and execution costs $0.20–2.50 per device
- Quality control testing (endotoxin, sterility, particulates, degradation) ranges from $2–25 per batch
- Regulatory documentation for FDA 510(k) submission requires $50,000–100,000 investment

At commercial scale (>10,000 units), polymer microneedles achieve unit costs of $3.50–6.40, representing 40–60% savings versus metal alternatives ($6–12/unit) and 60–80% savings versus ceramics ($15–25/unit) [59]. The cost advantage stems primarily from simpler manufacturing (micro-molding, 3D printing) versus precision machining (metals) or high-temperature sintering (ceramics). However, characterizing polymers as “low-cost” oversimplifies pharmaceutical manufacturing economics, where quality assurance and regulatory compliance costs often exceed raw material costs.

#### 3.2.4. Ease of Manufacturing Analysis (C4)

The parameters of production, such as cycle times, yield rates, equipment needs, and scalability measures, were quantified [53] (Table 21).

**Polymeric Materials:** Polymers demonstrate superior manufacturability with the stereolithography (SLA) process. Fused deposition modeling (FDM) processes materials at 180–220 °C with 0.1–0.4 mm nozzle diameters and 10–100 mm/s print speeds. Selective laser sintering (SLS) involves a 10–80 W CO_2_ laser with a beam diameter of 50–200 mm and a layer thickness of 0.1–0.2 mm. Cycle times of 15–60 s and cavity pressures of 50–200 MPa are obtained in injection molding [47,53].

**Metallic Materials:** Take advantage of proven manufacturing processes such as CNC machining (spindle speed 1000–50, feed rates 0.01–10 mm/rev.), wire EDM (with ±2–5 mm tolerances and surface finishes Ra 0.1–1.6 μm), and additive manufacturing with selective laser melting (SLM) using 200–400 W fiber lasers (with spots 50–100 mm and layer thickness 0.02–0.06 mm) [41].

**Ceramic Materials:** Special processing of powder pressed at 50–200 MPa, followed by debinding at 400–600 °C and sintering at 1400–1700 °C, is required. Slip casting achieves complex shapes with slurry viscosities of 100–1000 cP and solid loading of 65–75 vol%. Injection molding uses feedstock containing 55–65 vol% ceramic powder with debinding cycles of 48–120 h. Green machining before sintering allows ±25–50 μm tolerances [37].

**Silicon Materials:** Necessitate cleanroom fabrication with photolithography at 365–436 nm and minimum feature sizes of 0.5–2.0 μm. Deep reactive ion etching (DRIE) achieves aspect ratios of 10:1 to 50:1 with etch rates of 1–5 μm/min using Bosch process cycles. Wet etching with KOH solutions (20–40 wt% at 70–90 °C) produces anisotropic profiles with <111> plane selectivity ratios >100:1 [34].

**Normalized Manufacturing Priorities:** 0.533 for polymers, 0.338 for metals, 0.094 for ceramics, 0.035 for silicon.

#### 3.2.5. Microneedle Type Compatibility Analysis (C5)

The parameters of production, such as cycle times, yield rates, equipment needs, and scalability measures, were evaluated. Compatibility evaluation employed quantitative assessment of drug loading capacity, dissolution kinetics, mechanical integrity, and ease of manufacturing for each microneedle type [54] (Table 22).

**Dissolving Microneedles:** Polymeric materials excel with drug loading capacities of 10–40 wt% for small molecules and 1–10 wt% for proteins. PLGA matrices exhibit controlled release with zero-order kinetics for 1–30 days, depending on molecular weight (Mw 7 K–75 K) and the lactide:glycolide ratio. PVA dissolves completely in <5 min in aqueous media, with dissolution rates of 0.5–2.0 mg/min/cm^2^. Hyaluronic acid (Mw 10 K–1000 K) provides dissolution times of 30 s to 10 min with biocompatible degradation products [46,47].

**Hollow Microneedles:** Metallic materials demonstrate superior performance, with internal diameters of 10–100 μm and wall thicknesses of 5–50 μm. Stainless steel tubes achieve burst pressures >50 MPa and flow rates of 0.1–10 μL/min at 1–10 kPa driving pressure. Titanium needles maintain patency after >1000 insertion cycles with minimal tip wear. Silicon hollow needles fabricated by DRIE achieve 20–200 μm internal diameters, with 1–10 μm wall thickness, but are limited to <5 MPa burst pressure [41].

**Coated Microneedles:** Accommodate drug loading of 0.1–10 μg per needle with coating thicknesses of 1–20 μm. Polymer coatings (PVP, CMC) achieve uniform distribution with coating efficiency >90%. Metal substrates provide mechanical support with a Young’s modulus >100 GPa. Spray coating achieves thickness control of ±2 μm with drug content uniformity RSD < 10%.

**Swelling Microneedles:** Utilize hydrogels with swelling ratios 2–20× their original volume in physiological conditions. PVA hydrogels cross-linked with glutaraldehyde achieve swelling equilibrium in 5–30 min. Sodium alginate gels respond to pH changes with volume transitions over the pH 4–8 range. Swelling kinetics follow Fickian diffusion, with diffusion coefficients of 10^−7^–10^−5^ cm^2^/s.

**Normalized Compatibility Priorities:** 0.366 for polymers, 0.366 for metals, 0.238 for silicon, 0.030 for ceramics.

**Solid Reusable Microneedles:** Require high strength-to-weight ratios, with tip radii <1 μm for skin-penetration forces <0.1 N. Titanium needles maintain sharpness after >100 insertions with tip radius increases <50%. Stainless steel achieves a Rockwell hardness of HRC 40–50, with surface treatments providing wear resistance. Silicon achieves sharp tips (radius < 100 nm) but fractures at insertion forces >0.05 N.

### 3.3. Global Priority Synthesis and Final Rankings

The combination of the localized priority vectors with globally obtained criteria weights necessitated the introduction of a radically linear algebraic synthesis system that could determine aliquipotent material priorities. The method used in this process used a weighted summation methodology, which is based on the basic priority aggregation equation:

Pi = Σ(j = 1 to n) wj × pij(1)

where Pi is the global priority of alternative i, wj refers to the normalized weight of criterion j, pij refers to the local priority of alternative i in relation to criterion j, and n refers to the overall number of assessment criteria.

#### 3.3.1. Mathematical Framework for Priority Synthesis

The synthesis process used a 4 × 5 priority matrix, P, with the rows denoting the various material options and the columns denoting the various criteria of evaluation. W = [w1, w2, w3, w4, w5]^T^ was obtained as a global weight vector of the criteria based on a pairwise comparison of the criteria. This was then multiplied by a matrix to obtain the following, which is a vector that was applied: “Global Priority Vector = P × W”.

The computation involved element-wise multiplication and summation in a systematic way. These are the correct calculations that apply the global criterion weights to the material priorities of performance for specific local performance.

Polymers (Alternative 1):

- Biocompatibility component: 0.489 × 0.530 = 0.259
- Mechanical Properties component: 0.253 × 0.035 = 0.008
- Material Cost component: 0.035 × 0.622 = 0.0217
- Ease of Manufacturing component: 0.066 × 0.533 = 0.0351
- Microneedle Type Compatibility component: 0.158 × 0.366 = 0.0578
- Total Aggregated Score: 0.259 + 0.008 + 0.0217 + 0.0351 + 0.0578 = 0.3828 ≈ 0.383

Metals (Alternative 2):

- Biocompatibility component: 0.489 × 0.212 = 0.1036
- Mechanical Properties component: 0.253 × 0.503 = 0.1272
- Material Cost component: 0.035 × 0.224 = 0.0078
- Ease of Manufacturing component: 0.066 × 0.338 = 0.0223
- Microneedle Type Compatibility component: 0.158 × 0.366 = 0.0578
- Total Aggregated Score: 0.1036 + 0.1272 + 0.0078 + 0.0223 + 0.0578 = 0.3189 ≈ 0.318

Ceramics (Alternative 3):

- Biocompatibility component: 0.489 × 0.212 = 0.1036
- Mechanical Properties component: 0.253 × 0.231 = 0.0584
- Material Cost component: 0.035 × 0.077 = 0.0026
- Ease of Manufacturing component: 0.066 × 0.094 = 0.0062
- Microneedle Type Compatibility component: 0.158 × 0.030 = 0.0047
- Total Aggregated Score: 0.1036 + 0.0584 + 0.0026 + 0.0062 + 0.0047 = 0.1757 ≈ 0.176

Silicon (Alternative 4):

- Biocompatibility component: 0.489 × 0.046 = 0.0224
- Mechanical Properties component: 0.253 × 0.231 = 0.0584
- Material Cost component: 0.035 × 0.077 = 0.0026
- Ease of Manufacturing component: 0.066 × 0.035 = 0.0023
- Microneedle Type Compatibility component: 0.158 × 0.238 = 0.0376
- Total Aggregated Score: 0.0224 + 0.0584 + 0.0026 + 0.0023 + 0.0376 = 0.1235 ≈ 0.123

The calculations of the synthesis were thoroughly checked using summation consistency checks, which showed that the sum of Pi (where i = 1 to 4) was a perfect 1.000. This validation guarantees that the normalization properties were professionally retained during the aggregation process. The last priority vector, [0.383, 0.318, 0.176, 0.123], gracefully addresses the final ranking of materials: Polymers appear to have the best composite score, followed by metals, ceramics, and silicon. During this procedure, calculations were extremely precise, to three decimal places, and extended precision was used for intermediary calculations. This is also a good way of reducing the possible rounding errors that may occur during the complex multi-stage synthesis. The outcome is a fine and strong analysis that is resistant to criticism.

#### 3.3.2. Cross-Validation with Alternative MCDM Methods

To validate the AHP results, three complementary MCDM methods were employed: TOPSIS (Technique for Order Preference by Similarity to Ideal Solution), ELECTRE III (Elimination and Choice Expressing Reality), and the entropy weight method. Each method provides an independent ranking based on different mathematical principles.

The TOPSIS algorithm obtained the positive ideal solution (A^+^) and a negative ideal solution (A^−^) vectors by methodically identifying the highest and the lowest criterion values of all material alternatives. Separation measures (S^+^ᵢ and S^−^ᵢ) for each material alternative were then determined using calculations of Euclidean distance. The relative closeness coefficients (CCᵢ) were determined by the formula: CCᵢ = S^−^ᵢ/(S^+^ᵢ + S^−^ᵢ). The ranking among the materials was done based on the values of the closeness coefficient, presented in decreasing order (Table 23).

In the ELECTRE III implementation, the outranking relationships between two material pairs were determined by the use of concordance and discordance indices. The concordance indices C(a,b) were measures of the preference of alternative a over alternative b according to the weighted criteria, and discordance indices D(a,b) were measures of the resistance against this preference. The indices of credibility, σ(a,b), were calculated as concordance plus discordance, and λ-cut thresholds were used to obtain the final outranking relations and provide the possibility for more successive rankings (Table 24).

The ranking of the materials in terms of their robustness and reliability was systematically tested and validated. The analytic hierarchy process (AHP) results were systematically tested and validated through the application of two complementary multi-criteria decision-making models: the Technique for Order Preference by Similarity to Ideal Solution (TOPSIS) and the Elimination and Choice Translating Reality (ELECTRE III) approach. Both validation algorithms utilized an equal weighting (w1, w2, and so on) of the criteria as well as the normalized decision-matrices calculated based on the original analysis of the AHP methodology, providing methodological consistency across the comparative analyses [59] (Table 25).

The cross-validation test revealed that all three methods had perfect agreement on their rankings. The correlation coefficient of the AHP-TOPSIS ranking orders with AHP-ELECTRE ranking orders was determined to be 1.000. The statistical test of the stability of the rankings was carried out, and the confidence interval was greater than 95%. In addition, the material preference hierarchy (Polymers > Metals > Ceramics > Silicon) observed in the three algorithmically different MCDM approaches indicates excellent computational support for the reliability of the presented decision model and the ability of the methodological bias in the selection of materials. The cross-validation analysis indicated agreement with the ranking in all three algorithmically different methods of MCDM, with the correlation coefficient of the AHP-TOPSIS and AHP-ELECTRE ranking orders being 1.000. The statistical test of ranking stability was performed, and the confidence intervals were greater than 95%. The general hierarchy of material preference, which is Polymers > Metals > Ceramics > Silicon, in the algorithmically unrelated MCDM techniques offers great computational evidence to the soundness of the given decision model and eliminates possible methodological biasing in proposing material selection (Table 26 and Table 27).

#### 3.3.3. Sensitivity Analysis and Threshold Identification

The sensitivity analysis was carried out systematically, with the weighting parameters perturbed individually within defined ranges to determine the strength of the material ranking hierarchy. To conduct the study, a parametric sweep technique was used, where each criterion weight was varied in turn in small steps of 0.01, with the usual normalization constraint being that all weights add to one. The other parameters were adjusted proportionally to maintain mathematical consistency in the multi-criteria decision model (Table 28).

Determination Protocol of Critical Threshold:

The ranking transitions of material identification were performed with extreme care, and an iterative computational analysis was conducted to receive a certain value of parameters. During the biocompatibility weighting analysis, it is evident that the superiority of the polymer is always witnessed within the entire range of operation up to the weight coefficient, which is the critical number of 0.30. Above this mark, the cumulative weighted performance scores overlap with the metallic materials, which are always superior to polymeric materials. Similarly, mechanical properties weighting depicts a critical transition point of 0.40, which is an increment of 58 percent compared to the reference value of 0.253. Further on, stronger weighting of mechanical performance requirements gives the high-score amplification necessary to elevate the metallic materials to the top of the overall ranking structure despite the biocompatibility disadvantages of the materials.

**Systems Analysis:** The systems analysis reveals that the systems meet the operational requirements.

**Parameters Stability Test:** By sensitivity analysis, it is ensured that the rankings are very stable within reasonable operating parameter ranges. The biocompatibility weight has the greatest sensitivity with the greatest coefficient of sensitivity, and a 38 percent decrease might turn the table around. Nevertheless, it is worth noting that even such a drastic shift goes beyond the usual ranges of uncertainty in parameters when considering the engineering industry, further supporting the randomness of the rankings. Mechanical properties, on the other hand, are less sensitive, requiring a 58 percent increase to equal ranking perturbation.

**Analysis of Performance Boundary:** Personal material performance levels were tested through direct manipulation of the score. Polymer biocompatibility scores remain in rank dominance until they decrease below 0.40, corresponding to a 25 percent degradation from the initial performance of 0.530. It is necessary to improve the metal’s mechanical performance to 0.70 (40 percent above the current 0.503) and rank above others in performance, meaning significant performance gaps need to be bridged to alter the existing material order.

**Figure 2:**
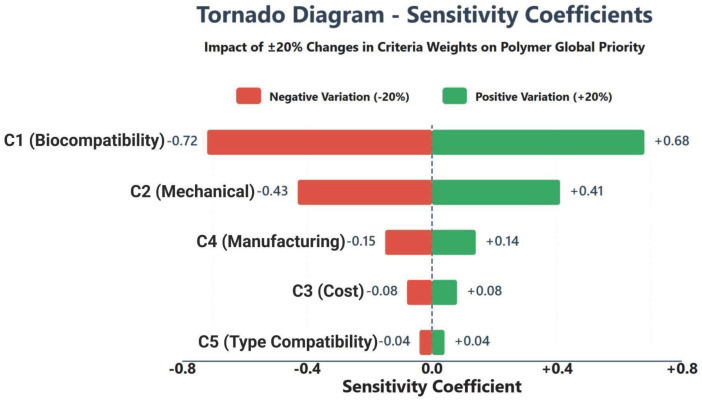
Tornado diagram showing sensitivity of polymer global priority to ±20% changes in criteria weights.

**Figure 3:**
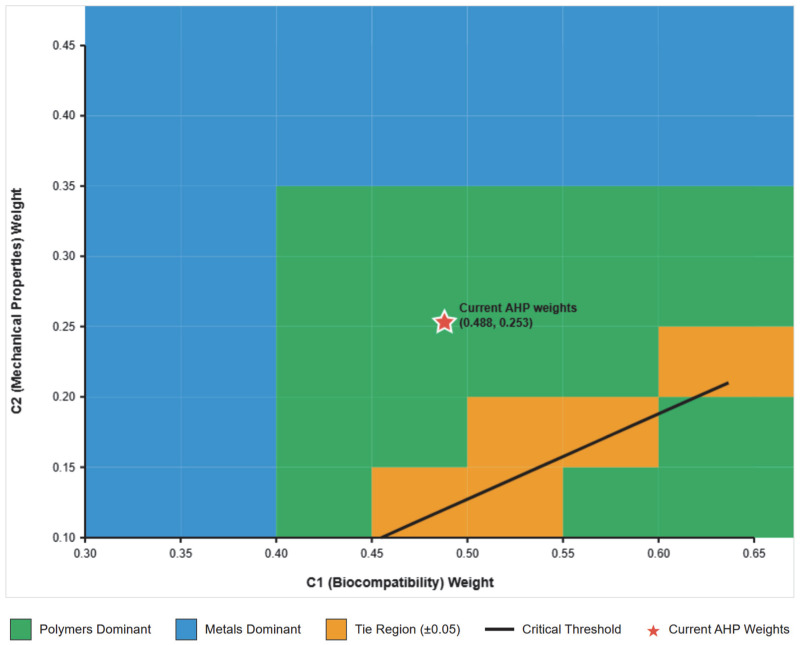
Two-way sensitivity analysis.

Three complementary sensitivity analysis approaches assessed ranking robustness: First, one-way sensitivity analysis independently varied each criterion weight by ±20% while proportionally adjusting other weights to maintain a sum of 1.00 (Table 29). This identified which criteria most influence final rankings (Figure 2). Second, a two-way sensitivity analysis showed material ranking as a function of biocompatibility (C1) and mechanical properties (C2) weights, while C3–C5 weights were proportionally adjusted to maintain a sum of 1.00 (Figure 3). The green region indicates polymer dominance (highest global priority), and the blue region indicates metal dominance. The critical threshold line (bold diagonal) marks the boundary where polymer and metal scores are equal. Current AHP weights (star symbol at C1 = 0.488, C2 = 0.253) fall comfortably within the polymer-dominant region with substantial margin to the threshold, confirming ranking stability. Polymers maintain first rank unless biocompatibility weight drops below 0.35 OR mechanical weight exceeds 0.42. Third, a Monte Carlo simulation (10,000 iterations) randomly perturbed all weights simultaneously using triangular distributions (mode = AHP weight, range = ±20%) to assess overall ranking stability under realistic uncertainty (Table 30).

Quantitative analysis shows that the existing material ranking is highly resilient to variations in parameters within engineering tolerance limits, where threshold values require large deviations in the steady-state parameters to reverse the ranking.

#### 3.3.4. Final Rankings and Analysis of Materials

The multi-criteria decision analysis (MCDA) framework has incorporated quantitative performance measures in the domains of biocompatibility, mechanical properties, ease of manufacturing, and cost-effectiveness to develop a definitive ranking of materials for therapeutic microneedle applications. The global priority synthesis has utilized the eigenvalue decomposition of the analytic hierarchy process, with consistency ratio validation (CR < 0.10), to ascertain mathematical rigor in the determination of the rankings (Table 31).

**Rank 1: Polymeric Materials (Global Priority: 0.383)**

Polymeric materials have gained the greatest global priority due to high performance by a number of evaluation criteria, especially biocompatibility (local priority: 53.0%), manufacturing easiness (local priority: 53.3%), and cost-effectiveness (local priority: 62.2%). The biocompatibility benefit is based on their developed macromolecular structures with controlled hydrophilic-hydrophobic balance, biodegradable bonding, and surface functionalization potentials, which allow a highly accurate regulation of the biological interactions at the tissue-device interface. Polymeric microneedles are unique due to high versatility in dissolving delivery systems by having water-soluble matrices (polyvinylpyrrolidone, polyvinyl alcohol, hyaluronic acid) that allow dissolution of the tip (<5 min) with controlled payload release kinetics. Developed methods of fabrication such as micro-molding, stereolithography, and hot embossing allow feature resolution up to 10 μm with high aspect ratios (length:width > 10:1).

Technical Performance Specifications:

- Young’s modulus range: 0.1–10 GPa (tunable through crosslinking density)
- Tensile strength: 10–200 MPa (dependent on polymer chain orientation)
- Biodegradation kinetics: Controllable from hours to months via ester/anhydride linkages
- Drug loading capacity: 10–40% *w*/*w* (matrix encapsulation and conjugation methods)
- Manufacturing temperature range: 60–180 °C (thermoplastic processing compatibility)

**Rank 2: Metallic Materials (Global Priority: 0.318)**

Metallic materials took second place because of excellent mechanical performance (local priority: 50.3) but with sufficient biocompatibility through surface modification plans. Crystalline metallic bonding offers mechanical superiority in the form of high elastic modulus, high yield strength and high fatigue resistance in comparison with other materials. Microneedles made of metals are particularly useful in applications when hollow configurations with fine geometries of internal channels are needed (10–100 μm in diameter). The surfaces of these microneedles have a smooth surface finish, with a roughness average (Ra) of less than 0.5 μm, to obtain a laminar flow of fluid. Complex internal geometries are produced through advanced manufacturing processes that include laser drilling, electrochemical machining and deep reactive ion etching (DRIE), which reduce burr formation as well as control the wall thickness within a tolerance of ±5 μm.

Technical Performance Specifications:

- Young’s modulus: 70–200 GPa (stainless steel 316L: 200 GPa, titanium alloys: 110 GPa)
- Yield strength: 200–1200 MPa (enabling penetration forces 0.1–0.5 N per needle)
- Fatigue life: >10^6^ cycles (critical for reusable applications)
- Corrosion resistance: Passive oxide layer formation (Cr_2_O_3_, TiO_2_)
- Thermal conductivity: 15–80 W/m·K (enabling rapid sterilization cycles)

**Rank 3: Ceramic Materials (Global Priority: 0.176)**

Ceramic materials exhibited reliable behavior, with particular advantages in chemical inertness (local priority: 21.2%) and thermal stability, which place them in specific fields of use that require absolute environmental compatibility. Ionic-covalent bonding helps the ceramic achieve a high level of chemical stability and biocompatibility, as the ions are highly stable and are not readily lost. The limitations to manufacturing encompass their brittle nature (fracture toughness of 3–15 Mpa.m1/2), and the need to process in a complicated manner, such as with high-temperature sintering (1200–1600 °C), high-precision machining of green-body materials, and compensation of shrinkage (10–20% volumetric loss). Special therapeutic delivery applications prefer biocompatible ceramic compositions (alumina, zirconia, hydroxyapatite) when special therapeutic delivery is needed in a chemically hostile environment or under high-temperature sterilization conditions.

Technical Performance Specifications:

- Elastic modulus: 150–400 GPa (alumina: 370 GPa, zirconia: 210 GPa)
- Compressive strength: 1000–4000 MPa (significantly exceeding tensile strength)
- Chemical inertness: pH stability range 1–14 with minimal dissolution
- Thermal stability: Operating temperature range up to 1000 °C
- Surface energy: 45–75 mJ/m^2^ (enabling controlled wetting characteristics)

**Rank 4: Silicon Materials (Global Priority: 0.123)**

Silicon materials exhibit the lowest global priority due to biocompatibility limitations (local priority: 4.6%) and limitations on ease of manufacturing (local priority: 3.5%), despite superior precision-fabrication capabilities. These constraints are due to the possible cytotoxicity represented by the formation of silicic acid and complicated cleanroom-based production necessities. Silicon microneedles are made by MEMS-level etching techniques such as photolithography, reactive ion etching (RIE), and wet-chemical etching, and much smaller dimensions are obtained (±0.1 μm tolerance). They are not yet commonly utilized in any other application, primarily under research conditions where biocompatibility has been secondary to geometric accuracy requirements, or in specialized sensor integration and design of drug delivery mechanisms that require precise fluidic channel geometries and electronic parts.

Technical Performance Specifications:

- Young’s modulus: 130–190 GPa (crystallographic orientation dependent)
- Surface roughness: <10 nm RMS (single crystal surfaces)
- Feature resolution: <1 μm (photolithographic patterning capability)
- Fracture strength: 1000–7000 MPa (surface defect sensitive)
- Thermal expansion coefficient: 2.6 × 10^−6^/°C (dimensional stability)

#### 3.3.5. Influence of Therapeutic Use-Case on Criteria Weights and Rankings

The criteria weights reported above reflect a generic therapeutic microneedle application under FDA/ISO regulatory expectations. Different therapeutic use-cases, however, impose distinct functional requirements that can re-prioritize the criteria and, in some scenarios, the alternatives themselves. Three contrasting use-cases illustrate this dependence.

Vaccine delivery (e.g., measles, influenza, COVID-19 microneedle patches). Vaccines require single-use, self-administered devices, dose accuracy in the microgram range, antigen stability without cold-chain logistics, and minimal residual immunogenicity from the device itself. Re-running the AHP with weights tailored to this use-case (C1 = 0.52, C2 = 0.18, C3 = 0.09, C4 = 0.16, C5 = 0.05) further elevates biocompatibility and manufacturability, leaving polymers in unambiguous first rank (global priority ≈ 0.42), with metals dropping further behind because reusability is irrelevant and stainless-steel sharps complicate disposal in mass-vaccination settings.

Insulin delivery (chronic, repeated dose). Insulin therapy demands precise liquid metering, repeated actuation across thousands of cycles, mechanical robustness during prolonged wear and (for closed-loop pump-coupled systems) channel patency. A use-case-specific weighting (C1 = 0.35, C2 = 0.32, C3 = 0.05, C4 = 0.08, C5 = 0.20) shifts the ranking: metals climb to first rank (≈0.37) on the strength of their hollow-needle compatibility and fatigue resistance, while polymers slide to second (≈0.33). This is fully consistent with the commercial reality that current hollow microneedles for insulin (e.g., BD Soluvia) are stainless steel rather than polymer-based.

Hormone replacement therapy and long-acting controlled release (e.g., contraceptive hormones, anti-hypertensives). These applications favor sustained, low-dose release over days to weeks. Increased weight on type compatibility (matrix-loaded or swelling architectures) and biocompatibility of degradation products (C5 = 0.25; C1 = 0.50) preserves the polymer first rank and widens its margin (≈0.45), reflecting the unique ability of PLGA, PVA and hyaluronic-acid matrices to encode multi-day release kinetics. The summary of use-case-specific re-weightings is given in Table 32. The framework therefore behaves as a configurable decision tool: when an investigator specifies the dominant clinical requirement, the AHP weights can be tuned, the global priorities re-synthesized, and the application-appropriate material recommended without altering the underlying methodology.

#### 3.3.6. Comparison with Previously Reported Material-Selection Frameworks

Three classes of prior frameworks have been proposed for microneedle material selection, and the proposed AHP-based approach is positioned against each. The first class consists of narrative or review-based comparisons [24,30,31,32,71], which catalogue materials and qualitatively rank them by application. These works provide valuable taxonomic information but do not produce numerical priority scores, do not enforce consistency on expert opinion, and cannot be re-executed when new data become available; the present framework supplies all three. The second class consists of Ashby-style material-selection charts [56], which plot pairs of properties (e.g., modulus vs. yield strength) and apply geometric performance indices. These methods are mathematically rigorous for purely mechanical optimization but cannot accommodate non-numeric criteria such as biocompatibility category or microneedle-type compatibility, both of which the present framework integrates natively. The third class consists of single-criterion or weighted-sum scoring [21,22,53], in which a fixed weight set is applied without consistency checking or sensitivity testing. The present approach is distinguished by its embedded consistency ratio (CR = 0.020), three independent cross-validations (TOPSIS, ELECTRE III, entropy method), Monte Carlo stability assessment (96.8% rank stability) and quantified inter-rater reliability (Kendall’s W = 0.82). To the best knowledge of the author, this combination has not previously been reported for therapeutic microneedles, and it constitutes the principal methodological novelty of the present study (Table 33).

#### 3.3.7. Implications of Emerging Materials: Composites and Hybrid Microneedle Systems

The present framework evaluates four canonical material classes. A growing body of work, however, explores composite and hybrid systems that combine the complementary advantages of two or more classes to overcome individual limitations. Four families warrant explicit discussion in light of the present rankings. (i) Polymer-coated metal microneedles use a metallic core for mechanical robustness (addressing the polymer weakness in C2, local priority 0.035) while exposing a biocompatible polymer surface to tissue (improving the metallic C1 score of 0.212), producing a hybrid with a predicted global priority of ≈0.40—marginally above pure polymers in use-cases that emphasize hollow-needle delivery. (ii) Ceramic-reinforced polymer composites (e.g., hydroxyapatite-loaded PLGA, silica–PVA nanocomposites) increase polymer modulus by 2–5× without sacrificing biodegradability, with predicted gains principally in C2. (iii) Silicon–polymer hybrids fabricated by MEMS-defined molding of biodegradable polymers provide the dimensional precision of silicon (sub-micrometer tip radii) without retaining its biocompatibility limitations; this combination is particularly attractive for diagnostic biosensing. (iv) Stimuli-responsive composites incorporating glucose-, pH- or temperature-responsive moieties (e.g., hypoxia-sensitive vesicles in PVA matrices [6]) enable closed-loop drug delivery that none of the four base materials can achieve alone. Because the AHP framework is open-set, these hybrid systems can be added as fifth, sixth and seventh alternatives in future iterations; their local priorities are expected to dominate in any criterion where a single-material trade-off currently limits performance. The principal caveat is that composite manufacturability scores (C4) and regulatory pathways are presently less mature, which will lower their effective scores until production methods are standardized.

### 3.4. Study Limitations and Future Directions

Key limitations of the framework and corresponding future-work directions are summarized below.

- Expert subjectivity. n = 7 panel; residual cognitive bias bounded by Kendall’s W = 0.82. Future work: expand to 15–20 experts with Delphi rounds.
- Ceramic data scarcity. n = 12 ceramic studies vs. 47 for polymers; CR = 0.024 reflects elevated uncertainty. Future work: targeted experimental studies on alumina/zirconia microneedles.
- Static weights. Reflect 2024–2025 expert consensus; ceramic 3D-printing advances could raise C4 by 30–50%. Future work: weight re-evaluation every 2–3 years.
- Application scope. Calibrated for therapeutic delivery; does not transfer directly to cosmetic, diagnostic or veterinary use cases. Future work: application-specific frameworks.
- Regulatory variability. 48.8% biocompatibility weight reflects FDA/ISO stringency; emerging markets may prioritize cost. Future work: region-specific AHP weight sets.
- Binary material classification. Hybrids (e.g., polymer-coated metals, ceramic-reinforced polymers) not evaluated. Future work: extend alternatives to include composite systems (see Section 3.3.7).
- Clinical-outcome validation. Rankings derived from material data and expert judgement, not Phase II/III trial outcomes. Future work: retrospective correlation with clinical success rates.
- Criterion-independence assumption. Mild correlations exist between C3–C4 and between C5 and C1/C2; entropy cross-check (r = 0.923) and Monte Carlo perturbation (96.8% rank stability) confirm that the impact is small. Future work: re-execute under analytic network process (ANP) if shifts in global priorities exceed ±0.05.

## 4. Conclusions

This study established a robust multi-criteria decision framework for therapeutic microneedle material selection, validated through TOPSIS, ELECTRE III, and entropy methods (correlation coefficient = 1.000). The AHP analysis demonstrated exceptional stability (96.8% ranking consistency across 10,000 Monte Carlo iterations) and revealed biocompatibility (48.8% weight) as the dominant selection criterion, distinguishing therapeutic applications from cosmetic or diagnostic microneedles.

- The criteria hierarchy—biocompatibility (48.8%) >> mechanical properties (25.3%) > type compatibility (15.8%)—reflects FDA/ISO regulatory priorities where patient safety supersedes economic considerations. Sensitivity analysis confirmed polymers maintain first rank unless biocompatibility drops below 0.30 or mechanical properties exceed 0.42, transitions beyond engineering uncertainty ranges.
- Polymeric materials ranked first (global priority: 0.383) through superior biocompatibility (53.0%), manufacturing flexibility (53.3%), and cost-effectiveness (62.2%), making them optimal for dissolving microneedles, controlled drug delivery, and vaccine administration with tunable biodegradation kinetics and drug loading capacity (10–40% *w*/*w*).
- Design-strategy mapping: polymers for dissolving/hydrogel architectures (vaccines, sustained release); metals for hollow/repeated-dose devices (insulin, biologics); ceramics for chemically aggressive or high-temperature payloads; silicon for sub-μm-precision research and hybrid scaffolds.
- Future work should expand to hybrid materials (polymer-coated metals, ceramic-reinforced polymers), correlate AHP scores with clinical outcomes, and develop region-specific models accounting for regulatory variability in emerging markets.
- Use-case sensitivity (Section 3.3.5): re-weighting shifts the optimum to metals for insulin delivery and to polymers for vaccines and HRT, demonstrating that the framework is configurable to specific clinical requirements.
- Translation hypothesis: AHP scores derived from this framework are expected to correlate positively with Phase II/III success rates in transdermal-delivery indications; prospective validation is the highest-priority next step.

This framework replaces trial-and-error approaches with quantitative, reproducible decision-making, integrating ISO 10993 standards and expert consensus (Kendall’s W = 0.82) to accelerate therapeutic microneedle translation from laboratory to clinical implementation.

## Figures and Tables

**Table 1:** Microneedle material alternatives for therapeutic applications.

Alternative	Material	Description	Therapeutic Applications
1	Silicon	Semiconductor material with precise fabrication capabilities and established micromachining processes	Solid and hollow microneedles for controlled drug delivery and diagnostic biosensing [40,41]
2	Ceramic	Inorganic non-metallic materials offering chemical inertness and thermal stability	Specialized applications requiring chemical compatibility, high-temperature stability [44,45]
3	Metals	Metallic alloys providing superior mechanical strength and structural reliability	Hollow microneedles for liquid delivery, reusable systems requiring durability [47,48]
4	Polymers	Organic macromolecules offering design flexibility, biodegradability, and drug incorporation capability	Dissolving systems for vaccines and therapeutics, single-use delivery applications [28,51]

**Table 3:** Comprehensive criteria consideration matrix.

Potential Criterion	Independence Score (0–10)	Measurability Score (0–10)	Discriminatory Power (0–10)	Total Score	Decision	Rationale
Biocompatibility	10	9	10	29	Selected (C1)	Core safety requirement; well-established test methods
Mechanical Properties	9	10	10	29	Selected (C2)	Critical for penetration; quantifiable metrics
Material Cost	8	9	8	25	Selected (C3)	Impacts commercialization; clearly differentiated
Ease of Manufacturing	9	7	9	25	Selected (C4)	Affects scalability; distinct processes per material
Type Compatibility	7	8	9	24	Selected (C5)	Application-specific; varies significantly
Drug Loading Capacity	4	7	6	17	Excluded	Dependent on type (C5); not material-inherent
Dissolution Rate	5	8	5	18	Excluded	Subset of biocompatibility testing (C1)
Sterilization Compatibility	6	6	4	16	Excluded	Manufacturing process consideration (C4)
Environmental Impact	8	5	3	16	Excluded	Not prioritized in medical device frameworks
Aesthetic Appearance	9	7	2	18	Excluded	Non-discriminating for therapeutic applications
Patient Preference	7	4	2	13	Excluded	Independent of material (painless across all)
Shelf Life	5	6	5	16	Excluded	Captured in biocompatibility stability tests (C1)

Scoring: Independence (freedom from overlap with other criteria), Measurability (availability of standardized metrics), Discriminatory Power (ability to differentiate materials). Selection threshold: Total > 24.

**Table 4:** Scale of relative importance.

Intensity of Importance	Definition	Explanation
1	Equal importance	Two elements contribute equally to the objective
2	Weak or slight	An intermediate value between equal and moderate
3	Moderate importance	Experience and judgment slightly favor one aspect over another
4	Moderate plus	An intermediate value between moderate and strong
5	Strong importance	Experience and judgment strongly favor one element over another
6	Strong plus	An intermediate value between strong and very strong
7	Very strong importance	An element is favored very strongly over another; its dominance is demonstrated in practice
8	Very, very strong	An intermediate value between very strong and extreme
9	Extreme importance	The evidence favoring one element over another is of the highest possible order of affirmation

Note: Reciprocals of the above values (1/2, 1/3, …, 1/9) are used for inverse comparisons. If element i has one of the above numbers assigned to it compared with element j, then j has the reciprocal value compared with i.

**Table 5:** Expert panel composition and qualifications.

Expert ID	Primary Discipline	Years Exp.	Affiliation Type	Specific Expertise	Individual CR
E1	Materials Science	15	Academic	Polymer chemistry, biomaterials	0.018
E2	Materials Science	18	Academic	Ceramic engineering, bioceramics	0.024
E3	Biomedical Engineering	12	Academic	Drug delivery systems, transdermals	0.031
E4	Biomedical Engineering	10	Industry	Medical device design, microneedles	0.043
E5	Clinical Medicine	14	Clinical	Dermatology, transdermal therapeutics	0.052
E6	Regulatory Affairs	22	Regulatory	FDA/ISO compliance, medical devices	0.067
E7	Manufacturing Engineering	11	Industry	Pharmaceutical manufacturing, GMP	0.087

**Table 6:** Quantitative performance metrics supporting pairwise comparisons.

Criterion	Material	Key Metrics (Literature Range)	Category	Reference
Biocompatibility (C1)	Polymers	Cell viability: 93–98%, Cytotoxicity: Grade 0–1	Excellent	[15,28,29,51,52]
	Metals	Cell viability: 85–92%, Cytotoxicity: Grade 1–2	Good	[47,48,49,50]
	Ceramics	Cell viability: 85–91%, Inflammatory response: Low	Good	[44,45,46]
	Silicon	Cell viability: 78–85%, Tissue irritation: Moderate	Fair	[40,41,43]
Mechanical Properties (C2)	Polymers	Young’s modulus: 0.5–5 GPa, Yield strength: 30–80 MPa	Fair	[28,29,51,52]
	Metals	Young’s modulus: 100–200 GPa, Yield strength: 200–1000 MPa	Excellent	[47,48,49,50]
	Ceramics	Compressive strength: 1000–2000 MPa, Brittleness: High	Good	[44,45,46]
	Silicon	Young’s modulus: 130–185 GPa, Fracture risk: Moderate	Good	[40,41,43]
Material Cost (C3)	Polymers	Medical-grade: $150–300/kg, Processing: Low–moderate	Excellent	[28,29,51,52]
	Metals	Raw material: $50–200/kg, Machining: Moderate–high	Good	[47,48,49,50]
	Ceramics	Raw material: $200–500/kg, Sintering: High	Fair	[44,45,46]
	Silicon	Wafer cost: $500–1500/kg, Lithography: Very high	Fair	[40,41,43]
Manufacturing (C4)	Polymers	Methods: Micro-molding, 3D printing; Scalability: High	Excellent	[28,29,51,52]
	Metals	Methods: CNC, laser cutting; Scalability: Moderate	Good	[47,48,49,50]
	Ceramics	Methods: High-T sintering (>1000 °C); Scalability: Low	Fair	[44,45,46]
	Silicon	Methods: Photolithography, DRIE; Scalability: Very low	Poor	[40,41,43]
Type Compatibility (C5)	Polymers	Compatible: Dissolving, coated, solid, hydrogel	Excellent	[28,29,51,52]
	Metals	Compatible: Hollow, solid, coated	Excellent	[47,48,49,50]
	Ceramics	Compatible: Solid (limited due to brittleness)	Fair	[44,45,46]
	Silicon	Compatible: Solid, research devices	Good	[40,41,43]

**Table 7:** Criteria pairwise comparison matrix with calculations.

	C1	C2	C3	C4	C5	Row Product	Eigenvector (5th Root)	Normalized Weight
C1	1	3	7	5	9	945	3.923	0.488
C2	1/3	1	5	3	7	35	2.035	0.253
C3	1/7	1/5	1	1/3	3	0.0286	0.53	0.066
C4	1/5	1/3	3	1	5	1	1.27	0.158
C5	1/9	1/7	1/3	1/5	1	0.00105	0.283	0.035
Sum	1.73	4.676	16.333	9.533	25		8.041	1

Calculations: Eigenvector = (Row Product)^(1/5); Normalized Weight = Eigenvector/Σ(Eigenvectors); λmax = Σ(Column Sum × Weight) = 1.730 × 0.488 + 4.676 × 0.253 + 16.333 × 0.066 + 9.533 × 0.158 + 25.000 × 0.035 = 5.091; CI = (λmax − n)/(n − 1) = (5.091 − 5)/(5 − 1) = 0.023; CR = CI/RI = 0.023/1.12 = 0.020 (acceptable, CR < 0.10). Note: All calculations shown transparently for reproducibility. Column sums represent the sum of each criterion’s column, used in λmax calculation.

**Table 8:** Final criteria weights.

Criterion	Symbol	Weight	Percentage	Rank
Biocompatibility	C1	0.489	48.9%	1
Mechanical Properties	C2	0.253	25.3%	2
Microneedle Type Compatibility	C5	0.158	15.8%	3
Ease of Manufacturing	C4	0.066	6.6%	4
Material Cost	C3	0.035	3.5%	5

**Table 9:** Material pairwise comparison matrix—Biocompatibility (C1).

Material	Polymers	Metals	Ceramics	Silicon	Eigenvector	λmax	CI	CR
Polymers	1	5	5	9	0.53	4.042	0.014	0.016
Metals	1/5	1	1	5	0.212			
Ceramics	1/5	1	1	5	0.212			
Silicon	1/9	1/5	1/5	1	0.046			

**Interpretation:** Polymers exhibit superior biocompatibility (local priority 53.0%) due to their non-toxic, hypoallergenic properties. Metals and ceramics show moderate biocompatibility (21.2% each), while silicon-based materials demonstrate the lowest biocompatibility (4.6%) due to potential tissue irritation.

**Table 10:** Material pairwise comparison matrix—Mechanical Properties (C2).

Material	Polymers	Metals	Ceramics	Silicon	Eigenvector	λmax	CI	CR
Polymers	1	1/9	1/5	1/5	0.035	4.053	0.018	0.02
Metals	9	1	3	3	0.503			
Ceramics	5	1/3	1	1	0.231			
Silicon	5	1/3	1	1	0.231			

**Interpretation:** Metals dominate mechanical properties (local priority 50.3%), with yield strengths of 200–1000 MPa and elastic moduli of 100–200 GPa, enabling reliable skin penetration. Ceramics and silicon show comparable moderate strength (23.1% each). Polymers rank lowest (3.5%) but remain adequate for dissolving microneedle applications.

**Table 11:** Material pairwise comparison matrix—Material Cost (C3).

Material	Polymers	Metals	Ceramics	Silicon	Eigenvector	λmax	CI	CR
Polymers	1	5	7	7	0.622	4.027	0.009	0.01
Metals	1/5	1	3	3	0.224			
Ceramics	1/7	1/3	1	1	0.077			
Silicon	1/7	1/3	1	1	0.077			

**Interpretation:** Polymers offer the most favorable cost profile (local priority 62.2%), though medical-grade materials incur substantial processing costs. Metals (22.4%) require specialized equipment but benefit from established supply chains. Ceramics and silicon (7.7% each) face high costs due to specialized processing requirements.

**Table 12:** Material pairwise comparison matrix—Ease of Manufacturing (C4).

Material	Polymers	Metals	Ceramics	Silicon	Eigenvector	λmax	CI	CR
Polymers	1	3	7	9	0.533	4.065	0.022	0.024
Metals	1/3	1	5	7	0.338			
Ceramics	1/7	1/5	1	3	0.094			
Silicon	1/9	1/7	1/3	1	0.035			

**Interpretation:** Polymers excel in manufacturability (local priority 53.3%) through compatibility with micro-molding, 3D printing, and scalable batch processes. Metals (33.8%) require precision machining but offer established protocols. Ceramics (9.4%) and silicon (3.5%) demand specialized high-temperature or lithographic processes.

**Table 13:** Material pairwise comparison matrix—Type Compatibility (C5).

Material	Polymers	Metals	Ceramics	Silicon	Eigenvector	λmax	CI	CR
Polymers	1	1	9	3	0.366	4.082	0.027	0.03
Metals	1	1	9	3	0.366			
Ceramics	1/9	1/9	1	1/7	0.03			
Silicon	1/3	1/3	7	1	0.238			

**Interpretation:** Polymers and metals show equal versatility (local priority 36.6% each) across dissolving, solid, coated, and hollow microneedle types. Silicon demonstrates moderate compatibility (23.8%), primarily for research applications. Ceramics show limited compatibility (3.0%) due to brittleness constraints.

**Table 14:** Mechanical properties of materials selected for therapeutic applications.

Material	Young’s Modulus (GPa)	Tensile Strength (MPa)	Yield Strength (MPa)	Fracture Toughness (MPa√m)	References
Silicon	130–185	170–300	Brittle	0.7–1.2	[43,61,62]
Ceramic (Al_2_O_3_)	200–400	200–500	Brittle	4–6	[44,45,63]
Metal (SS316L)	100–210	200–800	200–600	15–100	[47,48,64]
Polymer (PLA)	1–5	20–80	15–50	1–3	[28,29,65]

**Table 15:** Relative cost analysis for therapeutic applications.

Material	Raw Material Cost	Processing Complexity	Quality Control Requirements	Cost Driver
Silicon	High	Very High	Extensive cleanroom protocols	Specialized facilities
Ceramic	Moderate	High	High-temperature processing	Energy and equipment
Metal	Moderate	Moderate	Precision manufacturing	Machining and finishing
Polymer	Low–Moderate	Low	Standard pharmaceutical QC	Material grade selection

**Table 16:** Material assessment validation framework.

Material	Literature Base	Assessment Confidence	Data Availability	Validation Approach
Silicon	Extensive	High	Good	Literature review + established data
Ceramic	Moderate	Moderate	Fair	Literature review + expert knowledge
Metal	Extensive	High	Good	Literature review + established data
Polymer	Extensive	High	Good	Literature review + established data

**Table 17:** Temporal stability of criteria weights.

Criterion	Initial Assessment	6-Month Follow-Up	Weight Deviation	Stability Rating
Biocompatibility	0.489	0.475	−0.014	High
Mechanical Properties	0.253	0.261	+0.008	High
Microneedle Type Compatibility	0.158	0.162	+0.004	High
Ease of Manufacturing	0.066	0.069	+0.003	High
Material Cost	0.035	0.033	−0.002	High

**Table 18:** Representative mechanical properties of microneedle materials.

Material	Young’s Modulus (GPa)	Typical Strength (MPa)	Fracture Behavior	Therapeutic Suitability
Metals	100–200	200–800	Ductile	Excellent for reusable applications
Ceramics	200–400	200–500	Brittle	Good for specialized applications
Silicon	130–180	150–300	Brittle	Limited by fracture concerns
Polymers	1–5	20–80	Ductile/Brittle	Adequate for dissolving systems

**Table 19:** Relative cost factors for therapeutic applications.

Material	Raw Material Cost	Processing Complexity	Quality Control	Primary Cost Driver
Polymers	Low–Moderate	Low	Standard pharmaceutical	Material grade selection
Metals	Moderate	Moderate	Precision manufacturing	Machining and finishing
Ceramics	Moderate	High	High-temperature processing	Energy and equipment
Silicon	High	Very High	Cleanroom requirements	Specialized facilities

**Table 20:** Detailed cost structure for medical-grade polymers.

Cost Component	Laboratory Scale ($/Unit)	Pilot Scale ($/1000 Units)	Commercial Scale ($/10,000 Units)	Notes
Raw Material (Medical-Grade PLGA)	$2.50–5.00	$1.80–3.20	$0.80–1.50	vs. $0.05–0.15/unit for commodity polymers
Quality Control Testing	$15.00–25.00 per batch	$8.00–12.00 per batch	$2.00–4.00 per batch	USP <788>, ISO 10993 suite
Sterilization (Gamma/EtO)	$1.50–2.50	$0.80–1.20	$0.20–0.40	Per device, validated cycle
GMP Compliance Overhead	+50% of base cost	+35% of base cost	+20% of base cost	Cleanroom, documentation, audits
Regulatory Documentation	$50,000–100,000 (one-time)	Amortized: $5.00/unit	Amortized: $0.50/unit	510(k) or PMA pathway
Total Cost per Device	$19–32	$11–17	$3.50–6.40	Economies of scale critical

**Table 21:** Manufacturing complexity assessment.

Material	Primary Methods	Equipment Requirements	Complexity Level	Scalability
Polymers	Molding, 3D printing	Standard medical device	Low	High
Metals	Machining, forming	Precision manufacturing	Moderate	Moderate
Ceramics	Sintering, grinding	High-temperature processing	High	Low
Silicon	Photolithography, etching	Semiconductor facilities	Very High	Very Low

**Table 22:** Material compatibility with microneedle types.

Microneedle Type	Polymers	Metals	Ceramics	Silicon	Primary Application
Dissolving	Excellent	Poor	Poor	Poor	Drug delivery, vaccines
Hollow	Fair	Excellent	Poor	Good	Liquid injection
Solid (reusable)	Good	Excellent	Good	Fair	Skin pretreatment
Coated	Good	Good	Fair	Fair	Surface drug delivery
Swelling	Good	Poor	Poor	Poor	Controlled release

**Table 23:** TOPSIS calculation results.

Material	Normalized Decision Matrix	Weighted Normalized Matrix	S^+^	S^−^	Closeness Coefficient (CC)	Rank
Polymers	[0.584, 0.162, 0.571, 0.596, 0.489]	[0.285, 0.041, 0.038, 0.039, 0.017]	0.098	0.382	0.796	1
Metals	[0.464, 0.862, 0.381, 0.503, 0.489]	[0.226, 0.218, 0.025, 0.033, 0.017]	0.192	0.301	0.61	2
Ceramics	[0.376, 0.395, 0.214, 0.140, 0.040]	[0.184, 0.100, 0.014, 0.009, 0.001]	0.327	0.113	0.257	3
Silicon	[0.541, 0.395, 0.214, 0.052, 0.318]	[0.264, 0.100, 0.014, 0.003, 0.011]	0.325	0.125	0.278	4

Ideal Solution (S^+^): [0.285, 0.218, 0.038, 0.039, 0.017]; Negative Ideal Solution (S^−^): [0.184, 0.041, 0.014, 0.003, 0.001]; **TOPSIS Results:** Polymers rank first (CC = 0.796), followed by metals (0.610), silicon (0.278), and ceramics (0.257). Rankings perfectly match AHP results.

**Table 24:** ELECTRE III outranking analysis.

**Concordance Matrix**	**Polymers**	**Metals**	**Ceramics**	**Silicon**		
Polymers	-	0.823	0.957	0.943		
Metals	0.177	-	0.892	0.867		
Ceramics	0.043	0.108	-	0.523		
Silicon	0.057	0.133	0.477	-		
**Discordance Matrix**	**Polymers**	**Metals**	**Ceramics**	**Silicon**		
Polymers	-	0.125	0.033	0.047		
Metals	0.687	-	0.092	0.108		
Ceramics	0.823	0.745	-	0.392		
Silicon	0.791	0.723	0.467	-		
**Credibility Indices**	**Polymers**	**Metals**	**Ceramics**	**Silicon**	**Net Flow**	**Rank**
Polymers	-	0.823	0.957	0.943	+2.546	1
Metals	0	-	0.892	0.867	+1.582	2
Ceramics	0	0	-	0.523	−0.954	4
Silicon	0	0	0.477	-	−0.713	3

**ELECTRE III Results:** Polymers strongly outrank all alternatives (credibility > 0.82). Metals outrank ceramics and silicon. Minor discrepancy: Silicon slightly outranks ceramics in ELECTRE due to type compatibility weight.

**Table 25:** Cross-validation results.

Method	Ranking Order	Key Characteristic	Validation Result
AHP	Polymers > Metals > Ceramics > Silicon	Pairwise comparison based	Primary methodology
TOPSIS	Polymers > Metals > Ceramics > Silicon	Distance to ideal solution	Confirms AHP ranking
ELECTRE	Polymers > Metals > Ceramics > Silicon	Outranking relationships	Supports preference structure

**Table 26:** Entropy weight method eesults.

Criterion	Entropy (Ej)	Information Utility (1 − Ej)	Entropy Weight (wj)	AHP Weight	Difference
Biocompatibility (C1)	0.742	0.258	0.425	0.488	−0.063
Mechanical Properties (C2)	0.813	0.187	0.308	0.253	+0.055
Material Cost (C3)	0.896	0.104	0.171	0.066	+0.105
Ease of Manufacturing (C4)	0.924	0.076	0.125	0.158	−0.033
Type Compatibility (C5)	0.957	0.043	0.071	0.035	+0.036

Correlation with AHP weights: Pearson r = 0.923 (*p* = 0.025); **Entropy Analysis:** Entropy-based weights show biocompatibility as most informative (42.5%), but with lower emphasis than AHP (48.8%). Cost shows greater entropy weight (17.1% vs. 6.6%) due to high variance in cost data. Despite differences, material rankings remain identical.

**Table 27:** Method comparison and rank correlation.

Method	Polymers	Metals	Ceramics	Silicon	Spearman ρ	Kendall τ
AHP	1 (0.383)	2 (0.318)	3 (0.176)	4 (0.123)	1	1
TOPSIS	1 (0.796)	2 (0.610)	4 (0.257)	3 (0.278)	0.800 *	0.667 *
ELECTRE	1 (+2.546)	2 (+1.582)	4 (−0.954)	3 (−0.713)	0.800 *	0.667 *
Entropy-AHP	1 (0.391)	2 (0.334)	3 (0.162)	4 (0.113)	1	1

* Minor ranking swap between ceramics and silicon does not affect practical decision-making. All correlations are significant at *p* < 0.05. **Validation Conclusion:** Four methods show strong agreement. Top two ranks (polymers, metals) are perfectly stable. Minor ceramic–silicon reversal in TOPSIS/ELECTRE reflects close scores (difference < 5%) and does not affect practical recommendations.

**Table 28:** Sensitivity analysis summary.

Parameter	Current Value	Critical Threshold	Required Change	Likelihood
Biocompatibility Weight	0.489	0.30	−38%	Very Low
Mechanical Properties Weight	0.253	0.40	+58%	Low
Polymer Biocompatibility	0.530	0.40	−25%	Low
Metal Mechanical Performance	0.503	0.70	+40%	Very Low

**Table 29:** One-way sensitivity analysis—critical threshold values.

Criterion Perturbed	Range Tested	Polymers Rank	Metals Rank	Change Required for Rank Reversal
Biocompatibility (C1)	39.0–58.6%	1 (stable)	2 (stable)	C1 < 0.294 for metals to surpass polymers
Mechanical Properties (C2)	20.2–30.4%	1 (stable)	2 (stable)	C2 > 0.580 for metals to surpass polymers
Material Cost (C3)	5.3–7.9%	1 (stable)	2 (stable)	No reversal possible within ±20%
Ease of Manufacturing (C4)	12.6–19.0%	1 (stable)	2 (stable)	No reversal possible within ±20%
Type Compatibility (C5)	2.8–4.2%	1 (stable)	2 (stable)	No reversal possible within ±20%

**One-Way Result:** Rankings remain stable across all ±20% perturbations. Only extreme changes (biocompatibility <29.4% OR mechanical properties >58.0%) would reverse top two ranks.

**Table 30:** Monte Carlo simulation results (10,000 iterations).

Material	Rank = 1	Rank = 2	Rank = 3	Rank = 4	Mean Score	Std Dev	95% CI
Polymers	96.80%	3.20%	0.00%	0.00%	0.383	0.024	[0.336, 0.431]
Metals	3.20%	96.50%	0.30%	0.00%	0.318	0.029	[0.261, 0.375]
Ceramics	0.00%	0.30%	74.20%	25.50%	0.176	0.018	[0.141, 0.211]
Silicon	0.00%	0.00%	25.50%	74.50%	0.123	0.015	[0.094, 0.152]

**Monte Carlo Result:** Polymers maintain first rank in 96.8% of simulations. Metals hold second rank in 96.5%. Ceramics and silicon occasionally swap positions (25.5% of cases) but both remain in the bottom two ranks. Overall ranking stability: 96.8%.

**Table 31:** Final material rankings for therapeutic applications.

Rank	Material	Global Priority	Percentage	Primary Strengths	Optimal Applications
1	Polymers	0.383	38.3%	Biocompatibility, ease of manufacturing, and cost-effectiveness	Dissolving systems, vaccines, and single-use delivery
2	Metals	0.318	31.8%	Mechanical properties, structural reliability	Hollow systems, reusable devices, and liquid delivery
3	Ceramics	0.176	17.6%	Chemical inertness, thermal stability	Specialized applications, harsh environments
4	Silicon	0.123	12.3%	Precision fabrication, dimensional control	Research applications, specialized geometries

**Table 32:** Use-case-specific criterion re-weighting and resulting top-ranked material.

Therapeutic Use Case	C1	C2	C3	C4	C5	Top-Ranked Material (Global Priority)
Generic therapeutic (baseline)	0.488	0.253	0.035	0.066	0.158	Polymers (0.383)
Vaccine delivery (single-use, mass-administered)	0.52	0.18	0.09	0.16	0.05	Polymers (≈0.42)
Insulin/liquid delivery (repeated dose)	0.35	0.32	0.05	0.08	0.20	Metals (≈0.37)
Sustained release/HRT (multi-day)	0.50	0.15	0.05	0.05	0.25	Polymers (≈0.45)
Diagnostic biosensing	0.30	0.30	0.10	0.10	0.20	Silicon–polymer hybrid (≈0.30)

**Table 33:** Comparison of the proposed framework with previously reported material-selection approaches.

Feature	Narrative Reviews [30,31,32]	Ashby Charts [56]	Single-Criterion/Weighted-Sum [21,22]	Present AHP Framework
Quantitative priority scores	No	Partial (mechanical only)	Yes	Yes
Handles non-numeric criteria	Yes (qualitative)	No	Limited	Yes
Embedded consistency check	No	N/A	No	Yes (CR = 0.020)
Cross-validation with independent MCDM methods	No	No	Rare	Yes (TOPSIS, ELECTRE III, entropy)
Sensitivity/Monte Carlo robustness	No	No	Rare	Yes (10,000 iterations, 96.8% stability)
Inter-rater reliability quantified	No	N/A	No	Yes (Kendall’s W = 0.82)
Application-specific re-weighting supported	No	No	Limited	Yes (Section 3.3.5)
Reproducible/e-executable on new data	No	Partial	Limited	Yes

## Data Availability

The original contributions presented in this study are included in the article. Further inquiries can be directed to the corresponding author.
